# Improved Copper Circularity as a Result of Increased Material Efficiency in the U.S. Housing Stock

**DOI:** 10.1021/acs.est.1c06474

**Published:** 2022-03-18

**Authors:** Tong Wang, Peter Berrill, Julie Beth Zimmerman, Narasimha D. Rao, Jihoon Min, Edgar G. Hertwich

**Affiliations:** † Department of Chemical and Environmental Engineering, Yale University, New Haven, Connecticut 06520, United States; ‡ Center for Industrial Ecology, Yale University, New Haven, Connecticut 06520, United States; § International Institute for Applied Systems Analysis (IIASA), Schlossplatz 1, A-2361 Laxenburg, Austria; ∥ Yale School of the Environment, Yale University, New Haven, Connecticut 06520, United States; ⊥ Industrial Ecology Programme, Department of Energy and Process Engineering, Norwegian University of Science and Technology (NTNU), 7495 Trondheim, Norway

**Keywords:** copper circularity, housing service, use-phase material and energy demand, home renovation and improvement, material efficiency strategies, compromised environmental benefit

## Abstract

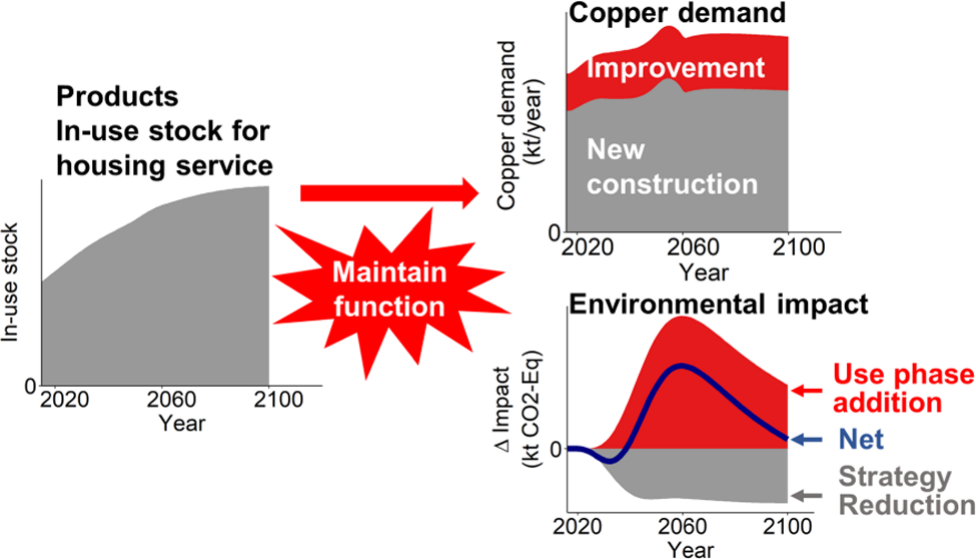

Material efficiency (ME) can support rapid climate change mitigation and circular economy. Here, we comprehensively assess the circularity of ME strategies for copper use in the U.S. housing services (including residential buildings and major household appliances) by integrating use-phase material and energy demand. Although the ME strategies of more intensive floor space use and extended lifetime of appliances and buildings reduce the primary copper demand, employing these strategies increases the commonly neglected use-phase share of total copper requirements during the century from 23–28 to 22–42%. Use-phase copper requirements for home improvements have remained larger than the demand gap (copper demand minus scrap availability) for much of the century, limiting copper circularity in the U.S. housing services. Further, use-phase energy consumption can negate the benefits of ME strategies. For instance, the lifetime extension of lower-efficiency refrigerators increases the copper use and net environmental impact by increased electricity use despite reductions from less production. This suggests a need for more attention to the use phase when assessing circularity, especially for products that are material and energy intensive during use. To avoid burden shifting, policymakers should consider the entire life cycle of products supporting services when pursuing circular economy goals.

## Abstract 2

Hereto overseen, the substantial demand for copper for renovation and home improvements has made it challenging to achieve a circular flow of copper in the U.S. residential sector.

## 1 Introduction

Material efficiency (ME), “providing material services with less material production and processing” following the definition by Allwood et al.,^1,2^ is an indispensable part of the rapid actions required to meet climate mitigation goals.^3−5^ It includes strategies like extending the lifetime of in-use products, more intensive product use, light-weighting, and material substitution.^1,2^ Circular economy (CE), an overlapping concept that aims at decoupling economic growth from material use, is attracting growing research interest and policy action globally.^6−14^ Material-use estimation from a service perspective allows for the assessment of demand-side ME strategies,^15−20^ and thus can aid in informing CE policies in terms of less primary material extraction and the system’s environmental impact. Although researchers increasingly urge for comprehensive circularity assessments to better inform options for resource management and sustainability,^9,11,13^ CE indicators for use-phase material use and environmental impacts are noticeably lacking.^21^ Products’ use phase influences the effectiveness of ME strategies, especially for products that require material or energy inputs to function.^22−24^

Copper offers superior electrical and thermal conductivity,^25^ and is an increasingly demanded material as a result of its massive use in buildings and rapidly growing use in clean energy and transportation.^26−30^ Meanwhile, copper has high vulnerability to supply restriction at the national level,^31,32^ and copper ore grade is declining.^33,34^ Primary copper production is energy intensive and has high environmental impacts, especially in human toxicity.^35^ Therefore, reducing the primary copper demand without compromising human welfare is of great interest. Currently, copper is used on average 1.9–2.1 times and for 47–60 years before final disposal.^14,36^ Increased recycling, regardless of the copper demand change by other ME strategies, is emphasized to alleviate environmental impacts while fulfilling societal services.^26,27^ The 10-year average copper recycling input rate (RIR, portion of the metal produced from scrap) globally is at 32%^25^ (varying from 20 to 50% across different geographical boundaries^25,37−40^) due to the limited end-of-life recycling rate (EoL-RR, percentage of a metal in discards that is actually recycled), increasing demand, and long lifetime of copper-containing products.^25,37,40−42^

Buildings account for around 50% of the current copper in-use stock^27,40^ and 28% of the 2019 copper use globally.^25^ Shelters and conditions for decent living require durable buildings^43^ and other major household appliances like air conditioners. The current research on material use in buildings often considers major structural components like roofs and external walls or other massively used materials like steel and concrete, while copper is often grouped together with other materials or considered partially.^20,44−46^ In the research from the copper perspective, the building and construction sector is commonly analyzed in detail as an independent and crucial category requiring copper, but the number of studies integrating/differentiating home improvement^47^ (all activities maintaining the function of in-use residential building stock, such as renovations and repair) are limited regardless of the research’s geographic boundary (e.g., globally or across regions like U.S., Europe, or China).^27,40,48,49^ Home improvement requires a distinct copper demand intensity and use patterns among building archetypes compared with new construction. In the future, home improvement will be increasingly important in that it could account for a higher share of the copper demand as residential buildings are aging and climate-based retrofitting is increasing.^50^ Therefore, it is crucial to understand the role of renovations and upgrades when investigating the future opportunities for copper recycling in the largest reservoir (building sector)^27,40,48^ of copper scrap to sustain long-term supply, which is still unknown.

It is noteworthy that trade-offs exist between material and energy efficiency (EE).^51^ For example, building retrofits can reduce operational phase greenhouse gas emissions but increase embodied emissions from material use.^46,52,53^ There is a small but growing literature on such trade-offs for household appliances and electronics.^22,23,54^ According to Boldoczki et al.,^23^ extending the lifetime of washing machines by 87% reuse in Germany reduces new production but only leads to 9% average impact reduction across various impact categories, including water consumption and land use, due to higher operational impacts. Reuse is more favored for appliances or electronics that are environmentally intensive in the production phase.^22,23,54^ In general, whether a ME strategy is preferred needs case-specific analysis.^22,23,54,55^ Some ME measures, such as more intensive use (e.g., reduced residential floor area per person), may face fewer trade-offs or offer synergies with other environmental dimensions.^51^ On the basis of previous studies, similar trade-offs between material efficiency and use-phase energy consumption can also be expected for copper ME strategies.

There are large uncertainties in the estimates of material use and associated environmental impact due to inconsistent material intensity (per unit material use) coefficients and wide ranges of lifetime.^56−59^ Material intensity has been estimated by calculating the ratio of economy-wide material consumption to gross domestic product,^60^ by referring to construction documents and on-site investigation,^61^ or by intensive literature review.^59,62^ The literature does not explicitly distinguish between material content (material actually embedded in products) and total material requirement (including all upstream demand), which might lead to underestimation of the total material demand and associated environmental impact. In the case of copper intensity (CI), total copper requirement (TCR) is the total demand of copper, including all of the upstream copper requirement for refined copper or copper semis materials; copper content (CC) is the copper embedded in products, which is useful in terms of calculating the current copper in-use stock and EoL scrap generation potential. About 11 and 16% of the copper used in the production of residential buildings (TCR) is not embedded in the buildings (as CC in products) due to losses during the initial-stage and final-stage manufacturing, respectively.^63^ Most new copper scraps collected from the manufacturing process are directly remelted.^64^ As for lifetime uncertainty, the literature estimates of the average U.S. residential building lifespan ranges from 61^57^ to 130^58^ years, and lifetime distributions are described by Weibull, Lognormal, or γ distributions.^57,58^ These losses and the uncertainty from the not fully understood lifetime distributions have not yet been well considered in copper recycling studies.

We hereby present a comprehensive framework to capture the flow of copper in the construction, maintenance, and end-of-life of residential buildings and to assess options to reduce primary copper use considering copper intensity and product lifetime uncertainty. In this paper, housing service was defined as the service provided together by residential buildings and major household appliances. It is noteworthy that this paper is an exploration of the potential of possible material efficiency strategies in reducing primary material use and influencing environmental impact under different future scenarios, rather than a prediction. We focused on the U.S. for a detailed analysis, and tested the framework by answering the following two questions:

(a) What is the potential of ME strategies to reduce the future primary copper demand and improve copper circularity to fulfill housing services in the U.S. considering uncertainties in copper intensity and lifetime distribution? Both capital formation of new buildings and appliances, and the maintenance of in-use stock in the form of home improvement are included. The modeling time period is 2015–2100.
(b) Using lifetime extension of refrigerators as an example, to what extent could the operational energy use of in-use stock for housing service influence the copper demand reduction and environmental benefit? Greenhouse gas (GHG) emission is used as the environmental indicator in this analysis and other indicators could be similarly adopted.

## 2 Methods

**Figure 1:**
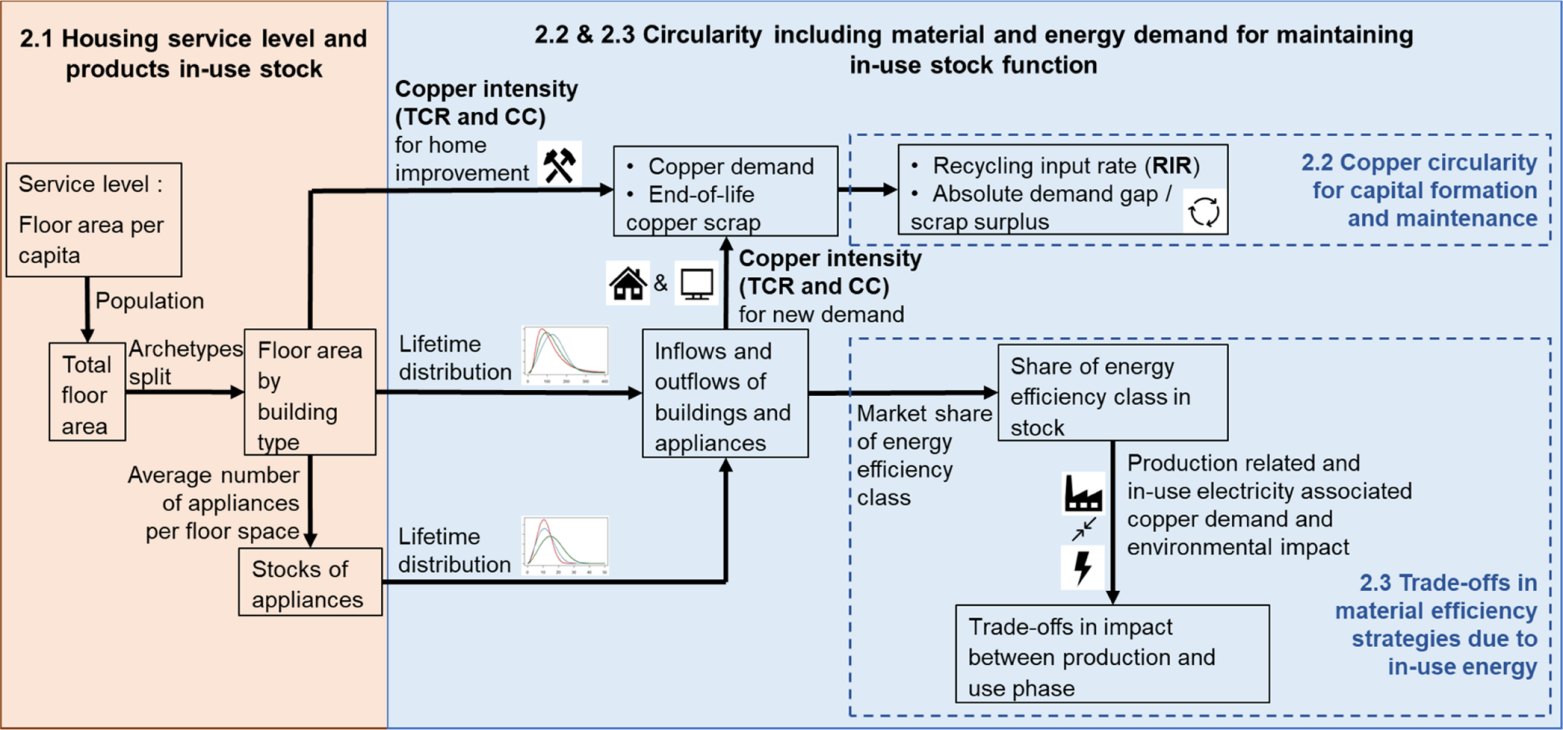
Overall framework of the methodology. The numbers on the figure are the corresponding method sections. TCR, total copper requirement; CC, copper content.

The overall framework to model copper circularity from the housing service perspective is shown in Figure 1. We used cutting-edge industrial ecology tools to address the following three parts in this framework sequentially: calculating housing service and required in-use stock of products, including three types of residential buildings—single-family (SFH), multifamily (MFH), and other residential structures, and major appliances (Section 2.1); assessing copper circularity for capital formation and maintenance under different ME strategies (Section 2.2); and identifying trade-offs in ME strategies due to operational energy use for in-use stock (Section 2.3).

### 2.1 Housing Service and Required Product Stocks

In-use stock of residential floor space was estimated based on future population, floor area per capita, and the market share of different building archetypes distinguished in the Resource Efficiency and Climate Change (RECC) framework.^4,20,65,66^ Following a what-if logic,^67^ the RECC framework defined parameters (e.g., service level, building archetypes) for 20 world regions subject to three storylines (low energy demand (LED),^68^ shared socioeconomic pathways SSP1 and SSP2^65^) and region-specific historical trends by identifying the existing scenario values in the literature, time-series regression analysis, or an expert consensus approach. We adopted the parameters of the U.S. for two scenarios, SSP2 and LED, from the RECC project. Appliance demand per floor space was estimated based on the 2015 Residential Energy Consumption Survey (RECS).^69^ See S-1 for details.

### 2.2 Copper Circularity for Capital Formation and Maintenance

Both capital formation (new residential buildings and major appliances) and maintenance (as home improvement) for housing services were considered in this paper. We identified the TCR and CC of the products and services supporting housing. Uncertainty relating to both copper intensity (Section 2.2.1) and products’ lifetime distributions (Section 2.2.2) were considered and incorporated into our model. A stock-driven dynamic material flow analysis^19^ (dMFA, in Section 2.2.3) was implemented to assess the in-use stock of products supporting housing services and then the inflows and outflows of products, and to further calculate the copper requirements based on product copper intensity. Copper scrap sources from end-of-life (EoL) products, manufacturing scrap (MS), and maintenance replacement (MR) were identified. The potential maximum recycling input rate (RIR)—the proportion of metal that can be produced from both production waste and postconsumer old scrap, the minimum amount of primary copper required, and possible surplus scrap under various scenarios—were assessed (Section 2.2.4).

#### 2.2.1 Copper Intensity (CI)

We differentiated two types of CI: TCR and CC. In this paper, the CI for new home construction and annual home improvement (in g/m^2^) were considered separately for single-family (SFH), multifamily (MFH), and other residential structures. Generally, TCR and CC were estimated per floor area by combining the copper use per monetary value, cost per building or the whole U.S. economy, and floor area. Copper use per monetary value was obtained by applying the waste input–output material flow analysis (WIO-MFA) method^70−72^ to the 2012 U.S. IO table^73,74^ based on Wang et al.^63^ Residential building prices (2019), consumer price indices of relevant economic sectors (2012 and 2019), home improvement costs (2019), mean floor area per housing unit (2019), and total housing unit (2019) were identified from the American Housing Survey^47^ and Bureau of Economic Analysis (BEA).^75^ CI results were compared with values in the literature^28,59,76^ from various years and across regions. The CI for home improvement includes copper use in major improvement areas like replacement of built-in heating equipment and electrical wiring, and in routine maintenance like fixing light switches. Home improvement activity happens across all ages of residential building stocks, and there is no clear correlation shown between building ages and the house improvement cost.^47^ CI was kept constant for future years in the base-case scenario. A more detailed illustration is shown in S-3.

For household appliances supporting housing services, per unit TCR (in g/unit product) was calculated using ecoinvent database version 3.6,^77^ and CC (in g/unit product) was mainly obtained from the literature.^28,78^ The uncertainty of all CI values was assessed by setting the lower and higher values as 50 and 200% of the base-case average values, respectively. See S-3 for details.

#### 2.2.2 Lifetime Distribution

As the average U.S. residential building lifespan ranges from 61 to 130 years in the literature,^57,58^ we adopted the average lifetime of 100 years in the base-case scenario. Weibull distribution with a shape parameter of 2.63 as suggested by Ianchenko et al.^58^ was adopted in the base-case scenario. The uncertainty of ±40% of the average lifetime of residential buildings was assessed by keeping the same shape parameter and changing the scale parameter accordingly. γ and Lognormal distributions were also assessed. The appliances’ lifetime distributions were adopted from Wang et al.^79^ Lifetime distribution parameters are shown in Table S1. An uncertainty of ±20% of the average lifetime of appliances was assessed.

#### 2.2.3 Dynamic Material Flow Analysis

Following the stock-driven dynamic material flow analysis procedure,^19^ the annual demand for residential buildings and associated products supporting housing services was estimated.^17,57,80,81^ The age structure (i.e., distribution of the product stock by the year of construction or purchase) of the products supporting housing services in 2015 was used as the start point of the dMFA model. By combining with the lifetime distribution, cohorts of products supporting housing services in future years were estimated. The 2015 age files of residential buildings that track the in-use stock by age cohorts were adopted from the RECC framework.^17,65,80−82^ The 2015 age files of appliances and average number of appliances per floor space were estimated from RECS.^69^ Annual inflows were calculated as the sum of annual in-use stock increase and annual outflow. Copper requirements and scrap generation were obtained by combining the results of the products with the CI. Copper scrap sources were differentiated among EoL products, manufacturing scrap (MS), and maintenance replacement (MR). The copper scrap from MR each year was assumed to be equal to the copper content in annual home improvement. See detailed equations and illustrations in S-4.

#### 2.2.4 Potential Recycling Input Rate (RIR) and Demand Gaps/Scrap Surplus

Two material flow indicators are used to assess the circularity: potential RIR and demand gap/surplus. If the total scrap was more than the copper demand, surplus scrap was accumulated to later years to meet the future copper demand. The copper demand gap (also referred to as the copper circularity gap) is the absolute copper difference between the total demand and the total scrap available. According to the literature, the overall potentially recyclable rate of copper is around 95%, and for cooling equipment and electronics, copper can be 100% recyclable.^83^ In this paper, the potential maximum RIR was estimated as the ratio of the total copper scrap available to the total copper demand, which can be approached only if the efficiency in collection, separation, and other processing stages is largely improved. See S-5 for more detail.

### 2.3 Trade-Off between Material Use and Operational Energy Use

The reduction of copper use by extending the lifetime of energy-using products can lead to a higher operational energy use when the new product has a higher efficiency than the one it is replacing, because of either technological improvements over time or wear-and-tear in existing products.^22,23,51,54^ Supplying this additional energy will require copper, and cause emissions. In this section, we used the example of refrigerators to examine the trade-offs in copper demand and environmental impact from product lifetime extension.

The EE of the in-use stock of refrigerators was estimated by combining the inflows and outflows of the refrigerators in this study and market EE by cohort using the information from the U.S. Energy Information Administration (EIA).^84^ Due to data availability, weighted averages of the maximum annual energy use were estimated for refrigerator sales in all years. To model the uncertainty from EE market shares, we compared three situations: (a) EE no improvement—the weighted average of the EE of refrigerator sales does not change after 2012; (b) EE conservative improvement—the weighted average of the EE of refrigerator sales improves (annual electricity consumption declines) to 397 kWh/yr (the energy star standard in 2014^84^) by 2100; (c) EE ambitious improvement—the weighted average of the EE of refrigerator sales improves (annual electricity consumption declines) exponentially to 47 kWh/yr by 2100. See S-6 for detailed illustration.

The copper saving and reduced environmental impact due to lifetime extension were compared with the additional copper demand and environmental impact due to more electricity use; the net effect was then shown. The copper demand and GHG emission per unit of refrigerator and per kWh of low-voltage electricity generation were assessed by life cycle assessment (LCA) using ecoinvent 3.6.^77^ To assess the impact of CI increase in electricity generation in an anticipated future with higher shares of renewable electricity,^29,85−87^ two situations were compared: (a) Electricity TCR no change—TCR per kWh of electricity keeps constant through the century; (b) Electricity TCR increase—TCR per kWh of electricity increases linearly from 0.234 g/kWh in 2015 to 0.640 g/kWh in 2100. To assess the impact of GHG emission intensity decline on electricity generation in a renewable future, two situations were compared: (a) Electricity GHG no change—GHG emission per kWh of electricity keeps constant through the century; (b) Electricity GHG decline—GHG emission per kWh of electricity declines rapidly to 0 by 2100. See S-6 for details.

### 2.4 Scenarios

Four scenarios were assessed and compared, including the base case and three ME strategies.

(a) Base-case scenario: service level (floor area per capita) and archetypes shares follow the SSP2 storyline as interpreted in RECC; lifetime distributions are base-case values.
(b) Scenario 1: Strategy 1—lifetime extension after 2020 (LT2020). The average lifetime of residential buildings and appliances after 2020 is doubled (for residential buildings, the average lifetime is extended from 100 years to 200 years, which also reduces the number of buildings that are replaced in the modeling period). Other parameters are the same as for the base case.
(c) Scenario 2: Strategy 2—service level stable after 2020 (SL2020). Other parameters are the same as for the base case.
(d) Scenario 3: Strategy 3—service level and archetypes shares follow the LED storyline as interpreted in RECC (SL_LED), where the utilization intensity of housing space increases from 67 m^2^/capita in 2015 to 38 m^2^/capita by 2060. Other parameters are the same as for the base case.

## 3 Results

Combining cutting-edge industrial ecology tools and various data sources, copper circularity from a housing service perspective, including copper demand and electricity use to maintain the function of in-use products, was assessed. Here, we present the results under various ME strategies considering both lifetime distribution and CI uncertainty. The results of in-use stock of products, annual demand for new products, and annual EoL products providing housing services are shown in Figures S2–S4.

### 3.1 Copper Use for Housing Services

**Figure 2:**
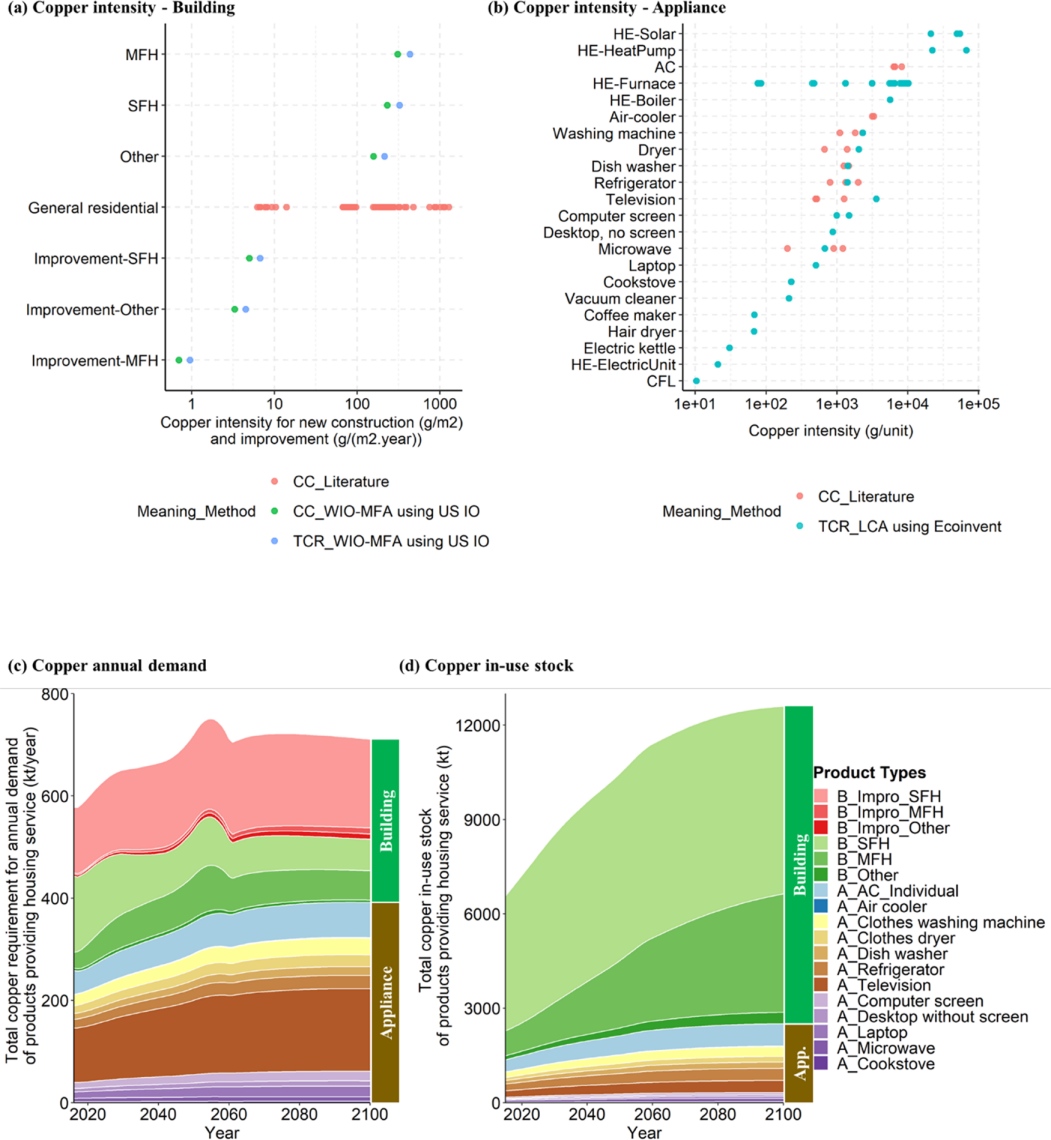
Copper use for housing services in the U.S. under the base-case scenario. (a) Copper intensity (CI) including both total copper requirement (TCR) and copper content (CC) for new home construction and yearly home improvement of existing stock. (b) CI for household appliances. (c) Annual copper demand for products supporting housing services in the U.S. (d) Total in-use Cu stock for housing services in the U.S. (App. means appliances). The items of (a) and (b) on the *y* axis are ranked by the median value of their data set, including TCR and CC. SFH means single-family residential building; MFH means multifamily residential building, Other means other residential structures, HE represents heating equipment, AC represents air conditioner, CFL represents compact fluorescent lamp, B_Impro_ means home improvement, B_ means building category, and A_ means appliance category.

Figure 2a,b shows the values of CI of buildings and appliances. The estimated CI results for a new home construction using the WIO-MFA method are within the range of values for general residential structures in literature from different years and various regions ranging from 6.2 to 1281.3 g/m^2^ (CI values larger than 3000 g/m^2^ are excluded in Figure 2a).^28,59,76^ Yearly TCR values per floor area of the existing stock for home improvement were estimated to be 6.7, 1.0, and 4.5 g/(m^2^·year) for SFH, MFH, and other residential structures, respectively; similarly, CC values were 5.0, 0.7, and 3.3 g/m^2^, respectively. The remarkable home improvement CI differences between SFH and MFH are mainly induced by their different copper uses per monetary value and total costs. MFH has the largest CI in new home construction, but the least CI for yearly home improvement, demonstrating the necessity to consider the difference of annual maintenance copper demand among various in-use stock archetypes. For most appliances, TCR is larger than CC, which is reasonable as TCR includes all of the upstream copper requirements. The CI for built-in heating and cooling equipment is significantly higher than for other household appliances due to more use of copper as thermal/electrical conductors.

Figure 2c,d shows the detailed annual demand and in-use stock of copper for housing services under the base-case scenario. For copper in-use stock, both buildings and appliances show increasing values while MFH buildings account for the most growth. The growth trend of copper demand for products, especially for MFH, is curbed remarkably around 2055 (Figure 2c), mainly due to the flattening of service level and stabilization of the archetype share of MFH from the middle of the century (Figure S2 and S3). Appliances account for about half of the annual demand, with televisions, individual air conditioners, washing machines, and refrigerators being the most significant copper-demanding appliances. As shown in Figure 2c, the copper demand to maintain in-use residential building stock in the form of yearly home improvement is comparable to the total annual new construction demand (including SFH, MFH, and other). Home improvement accounts for 42–61% of the total requirements for residential buildings and 23–28% for the whole housing services, including both residential buildings and major appliances, with growing proportions toward the middle of the century and staying relatively stable afterward, indicating the necessity of considering home improvement such as maintenance and repair when assessing the circularity.

### 3.2 Copper Demand and Scrap Generation under Scenarios with Uncertainty

**Figure 3:**
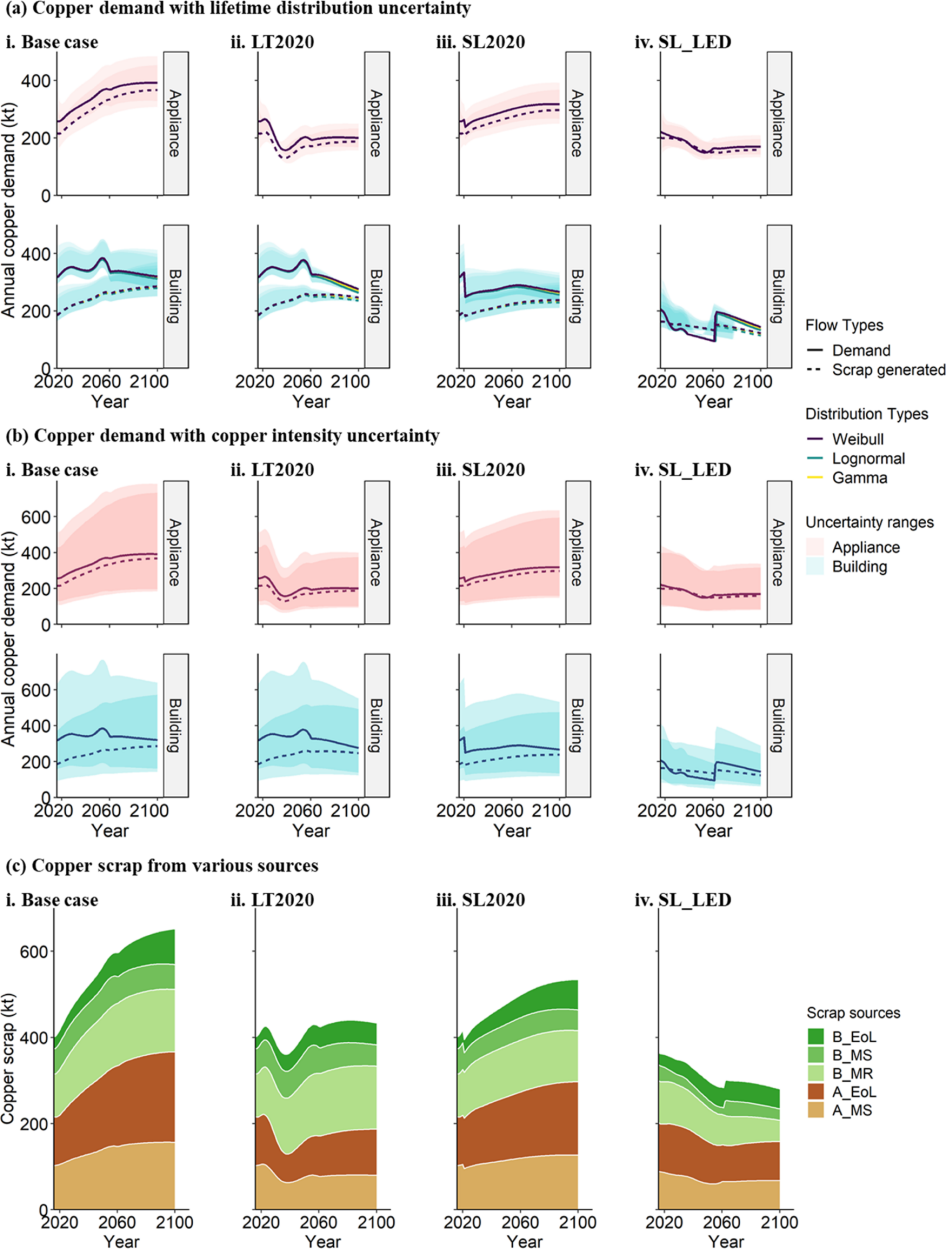
Annual copper requirement and scrap generation under four scenarios in the U.S. (a) Copper demand considering the uncertainty from lifetime distribution (buildings: Weibull, Lognormal, and γ distribution with average lifetime: ±40%; appliances: Weibull distribution with average lifetime: ±20%) under four scenarios. (b) Copper demand considering the uncertainty from copper intensity (50–200% of base value) under four scenarios. (c) Scrap from various sources under four scenarios. LT2020 represents Strategy 1—lifetime extension after 2020, SL2020 represents Strategy 2—service level stable after 2020, SL_LED represents Strategy 3—service level following the Low Energy Demand scenario,^17,68^ B_ means building category, A_ means appliance category, EoL represents end-of-life, MS represents manufacturing scrap, and MR represents maintenance replacement from home improvement.

The amount of copper in scrapped appliances is close to the demand for copper in new appliances (Figure 3a,b). Scrap from buildings can today cover two-thirds of the copper needed for new construction and repairs, and the demand gap for buildings is shrinking. All three ME strategies are effective in flattening/decreasing the annual copper demand for housing services, although there are large uncertainties arising from lifetime distributions and in particular CI for both buildings and appliances. It is noteworthy that, under the LT2020 strategy, scrap generation from the newly built buildings after 2020 slows down during the modeling period (by 2100), as well as the annual demand of buildings compared with the base case, keeping the in-use stock at the same level. Strategy 3, SL_LED, where service level follows the Low Energy Demand scenario, performs best in reducing the annual demand, where scrap generation in buildings even surpasses the demand in the middle of the century as residential building in-use stock decreases in the first decades (Figure S2). Home improvement makes 23–28, 23–42, 23–30, and 22–41% of the annual copper demand for base-case, LT2020, SL2020, and SL_LED scenarios, respectively. According to Figure 3c, total scrap generation is overall stable and decreasing for Strategies 1 and 3 (Figure 3c(ii,iv)), respectively. Although EoL scrap and manufacturing loss together contribute the most to the total scrap, home improvement replacement accounts for a considerable proportion, especially in the LT2020 strategy (25–36%).

### 3.3 Copper Circularity for Capital Formation and Maintenance

**Figure 4:**
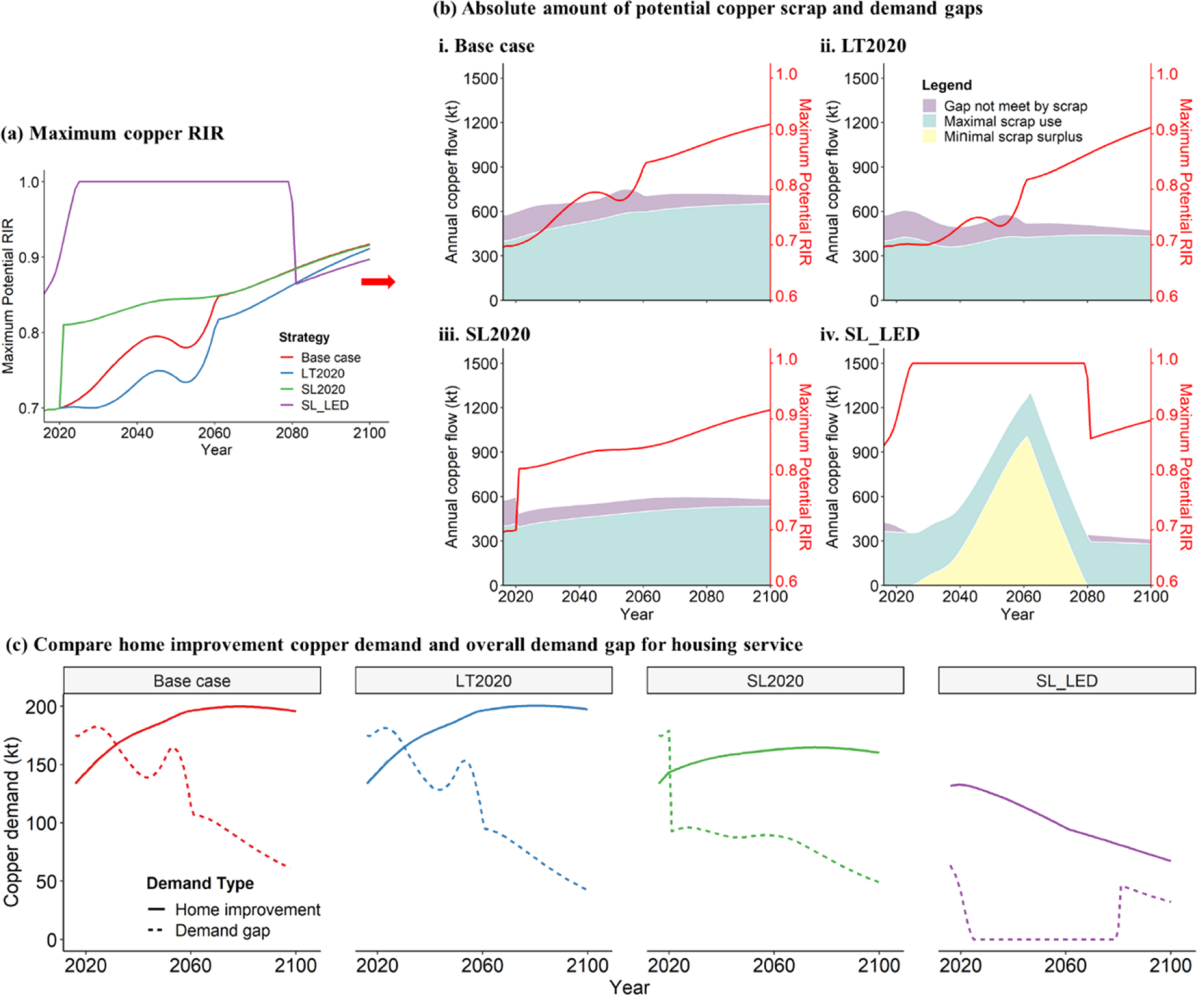
Circularity of copper in terms of the recycling input rate (RIR) and demand gap for housing service under four scenarios in the U.S. (a) Maximum copper RIR for housing service. (b) Overall copper demand gap/scrap surplus under four scenarios. (c) Comparison of home improvement copper demand and overall demand gap for housing service. LT2020 represents Strategy 1—lifetime extension after 2020, SL2020 represents Strategy 2—service level stable after 2020, and SL_LED represents Strategy 3—service level following the Low Energy Demand scenario.^17,68^

Given the narrowing of scrap generation and new product demand, the potential recycling input rates (RIRs) would increase in all scenarios (Figure 4). Overall, all three strategies reduce the potential minimum demand gap compared with base-case scenarios. However, the potential maximum RIR in LT2020 strategy is lower than base case as less scrap is generated when products are used longer. The SL_LED strategy, i.e., gradually reducing the floor space from 67 m^2^/capita in 2015 to 38 m^2^/capita by 2060 while keeping the same population growth as the base case, reduces the rate of new construction and the associated copper demand. Under the SL_LED strategy, total scrap (scrap generated in a specific year + scrap surplus from previous years) surpasses copper demand and accumulates in the middle of the century as the total in-use stock decreases in the first decades. Only after 2060 does a stable service level combined with continuous population increase the stock again (Figure S2). Although the potential demand gaps are consistently decreasing (Figure 4b), the copper demand and scrap generation from home improvement are increasing or remain stable except for the SL_LED strategy (Figure 3). Home improvement copper demand exceeds the demand gap of the overall housing service (including home improvement) for much of the next century (Figure 4c), limiting copper circularity in the U.S. housing services.

### 3.4 Compromised Environmental Benefit

**Figure 5:**
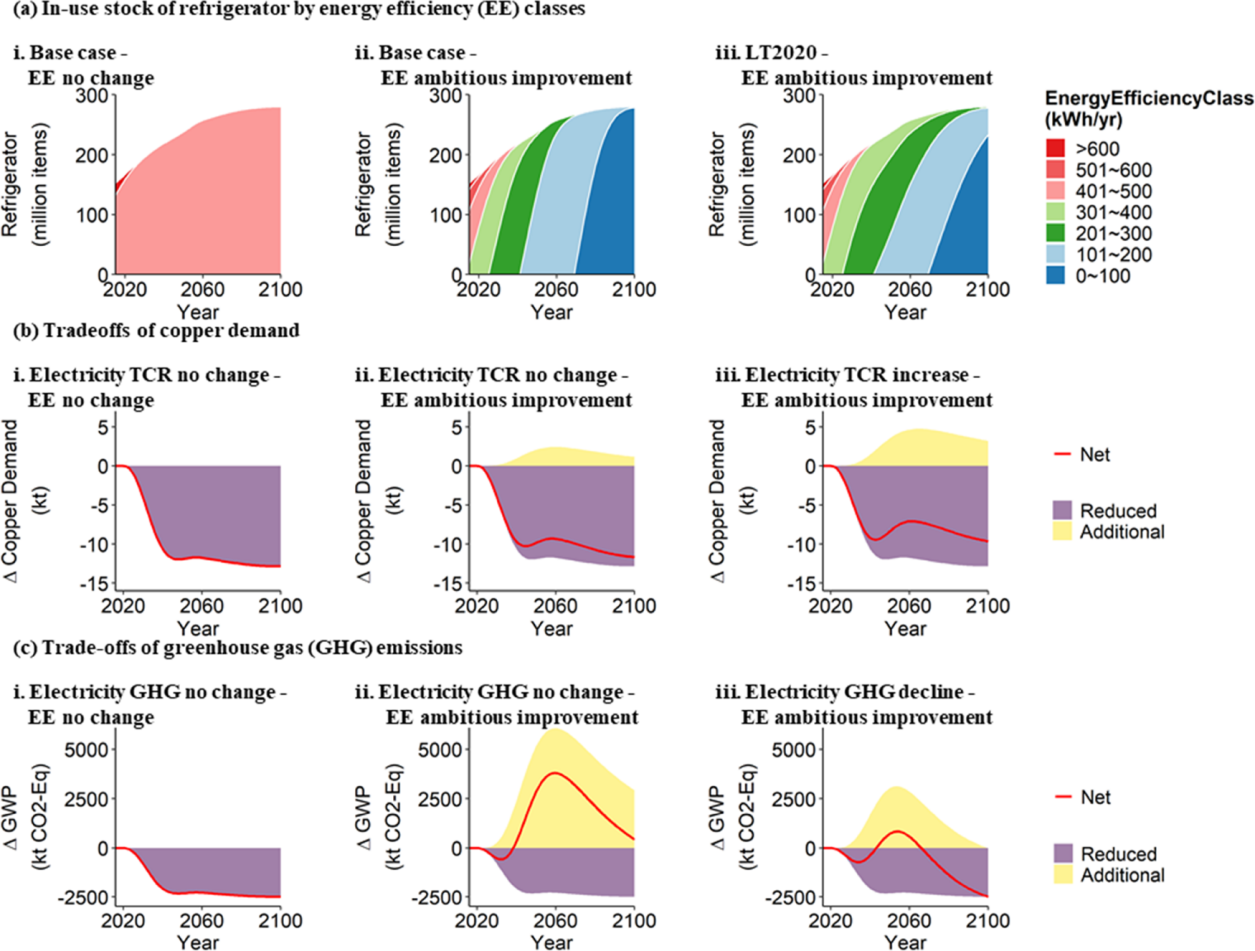
Lifetime extension-induced change of energy efficiency (EE) class composition and copper demand, and environmental impact related to the refrigerator demand to fulfill U.S. housing service. (a) Comparison of the in-use stock of refrigerators by EE classes between base case((a)-i, ii) and lifetime extension scenario((a)-iii) under two different EE improvement situations. (b) Trade-offs in copper demand between reduced production and additional electricity consumption by less-efficient refrigerators under different EE improvement and electricity total copper requirement (TCR) situations. (c) Trade-offs in greenhouse gas (GHG) emissions between reduced production and additional electricity consumption under different EE improvement and electricity GHG situations.

The trade-off between material efficiency and energy efficiency when extending the lifetime of refrigerators was analyzed under the LT2020 strategy (Figure 5). According to Figure 5a, lifetime extension increases the share of less-efficient refrigerators of in-use stock and decelerates the adoption rate of more energy efficient refrigerators, which is most evident under the situation that energy efficiency is improved ambitiously to 47 kWh/yr. As shown in Figure 5b(ii,iii), if TCR per kWh electricity is increased as in an anticipated renewable future and EE is improved ambitiously, additional copper demand due to higher use-phase electricity demand by low-energy efficient refrigerators is substantial compared with the reduced copper demand for production under LT2020 strategy. The benefit of lifetime extension in reducing GHG emissions (Figure 5c) is compromised remarkably and even reversed (more GHG emission). When new refrigerators are much more energy efficient than old refrigerators, the net impact on GHG emissions depends on the electricity mix (compare Figure 5(c)ii and 5(c)iii).

## 4 Discussion

This paper assessed the effectiveness of material efficiency strategies and emphasized the need to consider both material and energy demand to maintain the function of in-use stock while addressing circularity from the service perspective. Although reducing the service level, e.g., from 67 to 38 m^2^ per capita, has the largest potential to reduce the copper circularity gap for housing services, other material efficiency strategies, such as lifetime extension that does not affect the service level, could also decrease the annual copper use and shrink the primary copper demand. Although both more intensive floor space use and longer lifetime of appliances and buildings reduce the primary copper demand, use-phase requirement in the form of home improvement represents a substantial copper demand (22–42%) not affected by these strategies. Its relative importance increases compared with the base case and surpasses the demand gap for the overall housing service during most of the century. It thereby hinders the circular copper flow of the housing service. Therefore, finding ways to reduce the copper demand for home improvement must be a priority. Further, the environmental benefit of lifetime extension in the case of refrigerators can be eliminated entirely due to additional use-phase electricity demand by less-energy-efficient appliances. A quicker market penetration of highly efficient refrigerators can prevent such trade-offs.

Despite the high potential maximum RIR results after considering increasing stock implying a huge opportunity to increase the copper circularity by more recycling, it does not necessarily mean that the rate is currently achievable or would lead to lower environmental impacts. Not all copper products are currently recyclable,^83^ and the current global copper RIR is restricted by imperfect collection, separation, and processing efficiency in addition to the increasing demand.^41,64^ In addition to increasing the recycling efficiency, the recycling infrastructure needs to be expanded to accommodate more scrap. Moreover, scrap sources and grades should be dealt with differently. As recycling increases, the copper scrap grade decreases, and more energy consumption and environmental impact occur,^63^ an optimal recycling rate might exist.^88^ Limits to recycling in the case of sufficient supply of scrap were also found in the steel industry due to copper contamination.^89^ The maximum RIR for housing services does not imply that scraps are always kept for this single purpose, but rather is an exploration of the potential circular degree of copper flow to provide housing service if recycling practice (e.g., collection, separation efficiency) has been significantly improved.

The significantly lower copper requirement of home improvement in MFH could reflect a lower standard of in-house conditions. For example, low-income households and building owners of rental housing may spend less on maintenance/retrofits, and rating of electrical switches and circuitry could be lower in MFHs as they might have fewer and less powerful appliances. Therefore, floor area may be a crude representation of the service level. We used the average number of appliances per floor area to calculate the total demand of appliances and did not differentiate the copper intensity among appliance specifications, due to no reliable data being found on the actual variations in the demand and copper content of appliances by residential building types, across regions and in different years. For example, one large home could have the same number but a larger size of appliances compared with a small home. It is possible that the number of appliances does not increase linearly with the floor area. Furthermore, the actual changes during the long modeling period may not be fully captured in this paper. For example, future electricity is anticipated to be cleaner with lower GHG emissions per kWh, but higher copper demand in infrastructure due to the adoption of more renewable energy like wind and solar.^29,90^ Electricity from intermittent renewable energy like offshore wind requires grid expansion and thus needs more copper.^85−87^ Although high-voltage grids could potentially reduce the transmission loss and carry more power per cable (thus less copper), other issues like installation cost and thick insulation for safety need to be scrutinized.^91−93^

Uncertainty exists in the dynamics of copper intensity. On the one hand, with more awareness of climate mitigation, a shift to renewable energy is anticipated and demand for copper-intensive equipment like heat pumps and home charging stations for electric vehicles (EV) will increase. The share of new homes with heat pumps has increased from 23% in 2000 to 42% by 2020.^94^ Further growth is expected as deep renovations in pursuit of residential decarbonization could see the number of annual heat pumps installations for renovations reaching 6–8 million from 2030.^95^ The growth of residential solar photovoltaic (PV) installations is also strong; over 400 thousand residential PV systems were installed in 2020, up from 0.74 thousand in 2000.^96^ The remodeling of existing buildings to house more people would require copper, which might not be sufficiently caught in the model. On the other hand, dematerialization^97−99^ driven by substitution, sustainable consumption, technological transition, etc. reduces the copper intensity. Substitutes, like optical fibers for telecommunication wires and plastics (e.g., poly(vinyl chloride) (PVC)) for plumbing,^31^ can replace old applications and decrease the copper intensity in buildings. The substitutability for copper is high in plumbing, telecommunications, and ordnance, which together account for 21% of the total copper use in the U.S., but poor in electrical and electronics, which represent 38%.^31,100^ As long as copper intensity does not decrease substantially, the main conclusion of this paper addressing the importance of use-phase home improvement in achieving a circular copper flow in housing service would not change.

The system of this paper on copper in housing service is large but not independent. In addition to buildings, copper demand and scrap generation by other end-use sectors like transport and infrastructure are rapidly increasing,^28^ underlining the importance of coupling circularity improvement across sectors and over time. Trade-offs exist in ME strategies in addition to lifetime extension, for other services and beyond copper. For example, reducing the floor area per capita may increase the copper intensity per floor area, as one would expect approximately the same amount of frequently used appliances like refrigerators to be required on a smaller floor area, althouth they may not necessarily be of the same size. The total copper demand might decrease less than the dwelling size. Another example is in transport services: using lighter materials like aluminum instead of steel in vehicles could reduce the overall mass but increase the energy consumption in material production.^51^ Overall, our analysis highlights the necessity to consider the trade-offs in ME strategies among different materials and energy during the full life cycle. Furthermore, the rebound effect, i.e., total consumption change due to altered human behavior by economic variable change (e.g., lower price of smaller apartment or reused appliances),^101^ needs to be considered, as it might on the contrary increase the overall stock of products.^102,103^

## 5 Implications

This analysis demonstrates that including both material and energy demand for maintaining the function of in-use stock is necessary when assessing material circularity from a service perspective. It informs future circular economy policy to avoid burden shifting among life stages by revealing a more comprehensive picture about the effectiveness of circularity strategies while considering human wellbeing. For example, if future energy efficiency increases a lot, there could be a breakeven point beyond which policymakers should accelerate the turnover rate of certain products that are energy intensive in the use phase to take full advantage of energy efficiency improvements and increased scrap availability. Integrating material use and recycling information into integrated assessment models is a promising way to address future technology changes, which is still lacking.^104^ This framework could be used to address the circularity of other types of service like mobility in which case material efficiency strategies for materials used in transport systems like cobalt and lithium could be assessed.

## Supporting Information Available

Detailed description of methods; equations; data sources; glossary; and other informing results (PDF)
