# Supplementary material for: Improved Copper Circularity as a Result of Increased Material Efficiency in the U.S. Housing Stock
Source: Environ Sci Technol. 2022 Mar 18;56(7):4565–77. doi: 10.1021/acs.est.1c06474 (PMC8988293; doi:10.1021/acs.est.1c06474)
Supplement: Supplementary file 1 — es1c06474_si_001.pdf [file es1c06474_si_001.pdf]

Supporting Information

Improved Copper Circularity as A Result of Increased Material Efficiency in The US Housing Stock

*Tong Wang[1,2,3], Peter Berrill[2,4], Julie Beth Zimmerman[1,4], Narasimha D. Rao[3,4], Jihoon Min[3], Edgar G. Hertwich[5, *]*

[1] Department of Chemical and Environmental Engineering, Yale University, New Haven, Connecticut 06520, United States

[2] Center for Industrial Ecology, Yale University, New Haven, Connecticut 06520, United States

[3] International Institute for Applied Systems Analysis (IIASA), Schlossplatz 1 - A-2361 Laxenburg, Austria

[4] Yale School of the Environment, Yale University, New Haven, Connecticut 06520, United States

[5] Industrial Ecology Programme, Department of Energy and Process Engineering, Norwegian University of Science and Technology (NTNU), 7495 Trondheim, Norway. Phone: +47 97512109.

Email: edgar.hertwich@ntnu.no

Number of pages: 27

Number of figures: 6

Number of equations: 23

Number of tables: 3

S-1. Lifetime distribution and appliances per floor area

While the importance of lifetime distribution has been recognized in estimating product stocks, flows and associated environmental impact, large uncertainty range exists[1–4]. According to Aktas and Bilec[1], average US residential building has an average lifetime of 61 years with a 90% confidence range of 21~105 years. Ianchenko et al.[2] further provided an estimation of 130 years. In this paper, Weibull distribution with a shape parameter of 2.63 as suggested by Ianchenko et al.[2] was adopted in base case value. Uncertainty of +/- 40% average lifetime of residential building was assessed by keeping the same shape parameter and changing scale parameter accordingly. Uncertainty of distribution types including Gamma and Lognormal distributions were assessed. The appliances lifetime distributions were adopted from the 2005 values in Wang et al.[4] The differences between the 1995 and 2005 average lifetimes (|(1995value-2005value) | /2005value) in Wang et al.[4] for different appliances are 0~9.35% except for microwave which is 32.7%. For simplicity, we assessed the same uncertainty ranges of +/-20% for all chosen appliances. Lifetime distribution parameters under base case were shown in *Table S1*.

Table S1 Lifetime distribution and average demand of products supporting housing services per floor space

Products supporting housing service	Weibull parameters		Average number of appliance per thousand square meters in 2015 US based on RECS[5]
	Shape k	Scale λ	
Residential building	2.63[2] (Weibull distribution)	Scale parameter changes with average lifetime	-

	4.22[2] (Shape parameter for Gamma distribution)	Rate parameter changes with average lifetime	
	0.57[2] (sdlog parameter for lognormal distribution)	meanlog parameter changes with average lifetime	
Heating equipment-Heat pump	1.8[4]	15.8[4]	0.70 (per heated area); 0.61
Heating equipment-Others represented by solar	1.8[4]	15.8[4]	1.65 (per heated area); 1.44
Heating equipment-Central warm-air furnace	1.8[4]	15.8[4]	3.64 (per heated area); 3.18
Heating equipment-Steam or hot water system	1.8[4]	15.8[4]	0.47 (per heated area); 0.41
Heating equipment-Electric unit	1.8[4]	15.8[4]	1.68 (per heated area); 1.46
Air conditioner (AC)	2.8[4]	12.3[4]	8.68 (per cooled area); 5.94
AC-Individual Units	2.8[4]	12.3[4]	3.64 (per cooled area); 2.49
Air cooler	2.4[4]	13.6[4]	0.19 (per cooled area); 0.13
Washing machine	2.2[4]	13.9[4]	4.42
Dryer	2.6[4]	16.5[4]	4.30
Dish washer	1.6[4]	13.1[4]	3.61
Refrigerator	2.2[4]	16.5[4]	6.91
Television (TV)	2.1[4]	12[4]	12.34

Computer screen	2.5 [4] (Flat panel display monitors)	7.5 [4] (Flat panel display monitors)	2.65 (The same as Desktop)
Desktop without screen	2.1[4]	9.6[4]	2.65 (The same as Desktop)
Laptop	1.5[4]	5.2[4]	5.22
Microwave	0.8[4]	14.7[4]	5.36
Cookstove	2.5[4] (Kitchen appliances)	18.0[4] (Kitchen appliances)	5.00
Vacuum cleaner	1.5[4]	10.3[4]	-
Coffee maker	1.8[4]	7.9[4]	-
Hair dryer	1.3[4] (Personal care)	10.8[4] (Personal care)	-
Electric kettle	1.8[4]	7.9[4]	-
Compact fluorescent lamp (CFL)	2.1[4]	9.1[4]	-

S-2 Model assumptions

General settings/assumptions in the model:

- Time horizon: 2015~2100
- A stock-driven dynamic material flow analysis model[6] is used.
- Three types of US residential building archetypes are considered: single-family home (SFH), multifamily home (MFH) and other residential structures.
- Lifetime distributions for three types of US residential buildings are the same with a +/- 40% uncertainty range.
- Appliances demand per floor area is summarized in Table S1.
- Lifetime distributions for appliances are with a +/20% uncertainty range.

- Two types of copper intensity parameters are considered: total copper requirement (TCR) and copper content (CC).
- Copper intensities are differentiated among three types of residential buildings and appliances; they remain constant over time, with an uncertainty range of 50~200% of the base values

Table S2 Scenario specific assumptions

	Service level (floor area per capita)	Population	Archetypes shares	Lifetime distribution
Base case	Following SSP2 storyline as interpreted in the Resource Efficiency and Climate Change (RECC) framework[7–10]		Following SSP2 storyline as interpreted in RECC[7–10]	Table S1
Scenario 1 - lifetime extension after 2020	Same as base case	Following SSP2 storyline as interpreted in RECC[7–10]	Same as base case	Average lifetime of residential buildings and appliances after 2020 is doubled.
Scenario 2 - service level stable after 2020	Stable after 2020		Same as base case	Same as base case
Scenario 3 - Low energy demand (LED)	Following LED storyline as interpreted in RECC[7–10]		Following LED storyline as interpreted in RECC[7–10]	Same as base case

The SSP2 storyline represents a "Middle of the road" scenario following historical trends, whereas the LED storyline can be seen as a bottom line for service provision[10,11]. Although SSP1 is also included in the Resource Efficiency and Climate Change (RECC) framework[7–10], the major parameters used in the model (service level, population, archetypes shares) for SSP1 are

between SSP2 and LED storylines. Therefore, we think the chosen of SSP2 and LED could give us simpler and clearer results.

S-3. Copper Intensity

S-3.1 New home construction

According to Wang et al.[12], copper content (CC) per monetary unit of single-family (SFH), multifamily (MFH), other residential structures and home improvement (represented by residential maintenance and repair sector) in 2012 have been estimated to be 0.176, 0.095, 0.282 and 0.299 t/million USD, respectively; similarly, total copper requirement (TCR) were 0.247, 0.134, 0.384 and 0.405 t/million USD, respectively. Average house value for SFH, MFH and other residential structures in 2019 are about 334,090, 445,977 and 78,350 dollars[13]. Based on the consumer price index (CPI) in the US in 2012 (2012=100) and 2019 (135.385, 143.68, 122.834 and 117.379 for SFH, MFH, other residential structures and residential maintenance and repair, respectively)[14], house value in 2012 were estimated to be 246770, 310396, and 63785 dollars for SFH, MFH and other residential structures, respectively. Average floor area for SFH, MFH and other residential structures were 189, 96 and 115 square meters[13], respectively. CC per floor area were thus estimated to be 230, 308 and 156 g/m2, respectively; similarly, TCR were 322, 433, and 213 g/m2, respectively.

CI values were compared with values from literature review across regions from different years[15–17] ranging from 6.2 to 1281.3 g/m2 (CI larger than 3000 g/m2 were excluded in Figure 2 (a)).

S-3.2 Home improvement of existing stock

In this paper, we estimated CI per unit of building stocks for yearly home improvement based on TCR and CC results per monetary unit, home improvement cost, and total floor space. Total home improvement expenditures like replacement of built-in heating equipment and electrical wiring (except routine maintenance) are about 246.5, 5.5 and 7.3 billion USD in 2019; while routine maintenance costs like fixing light switches are about 68.1, 2.9 and 2.9 billion USD, respectively[13]. Therefore, based on CPI in 2012[14], total costs for home improvement including routine maintenance in 2012 were estimated to be 268.1, 7.1 and 8.7 billion USD, respectively. As total floor area in 2019 were 16.2, 3.0 and 0.8 billion square meters in the US, average TCR per floor area of total existing stock for home improvement were estimated to be 6.7, 1.0 and 4.5 g/m2 for SFH, MFH and other residential structures; similarly, average CC were 5.0, 0.7 and 3.3 g/m2, respectively.

S-3.3 Household appliances

CC were obtained from literature[16,18] for various household appliances. TCR is the total requirement of the process - “market for copper” per unit of appliances calculated using ecoinvent database version 3.6 [19]. The values from WIO-MFA method using IO tables were not used for appliances due to their low resolution. For example, in the US IO table[20,21], household appliances are only separated into household cooking appliance, refrigerator and freezer, laundry equipment and others; heating boilers and stoves are classified into heating equipment (except warm air furnaces); warm air furnace and heating pumps are both in “Air conditioning, refrigeration, and warm air heating equipment manufacturing”[22].

There is little information in literature on CI of heating equipment and heating equipment varies a lot in terms of types (furnace, heat pump, electric heater, etc.), heating fuel (natural gas,

electricity, wood, etc.) and rated capacity (in kW). For example, central warm-air furnace, heat pumps and boilers could all be used as main heating equipment according to residential energy consumption survey (RECS)[5] and the heat capacity of heating equipment could be 4 kW or 10 kW according to ecoinvent database[19]. We matched the possible heating equipment between RECS and ecoinvent and calculated the TCR for different heating equipment: central warm-air furnace (furnace: 76 ~ 10203 g/unit); steam or hot water system (gas boiler or oil boiler: ~5642g/unit); heat pump (22304 ~ 66920 g/unit); others (solar collector system: 21065 ~ 54481 g/unit); electric unit (21 g/unit). Only the heating equipment with a capacity lower than 100 kW was considered to be used in residential buildings.

If only TCR or CC was available, the missing one was estimated based on the CC/TCR ratio obtained from Wang et al.[12] For example, CC was not available for PC related products or cookstoves; so for these products CC was estimated based on CC/TCR ratio of 0.54 and 0.66, respectively. TCR was not available for air conditioner (AC) and air cooler, thus was estimated based on the ratio of 0.90. If the average CC of an appliance was higher than its TCR, another CC was estimated to be used in base case calculation using CC/TCR ratio. According to the results, only average CC for microwave from literature was larger than TCR. Thus, we estimated CC based on the ratio of 0.74.

S-4. Dynamic material flow analysis model

The required products include residential floor space and appliances. In-use stock of product p in year t was denoted as $IUS_p(t)$. $IUS_p(t)$ was estimated separately for residential floor space ($IUS_{p-FS}(t)$) and appliances ($IUS_{p-A}(t)$). The in-use stock of residential floor space was calculated as follows:

$$IUS_{p-FS}(t) = SL(t) * POP(t) \quad (1)$$

where product p includes the provided residential floor space by single family house (SFH), multifamily house (MFH) and others; SL(t) represents service level - floor space per capita - in year t; POP(t) represents population in year t.

Appliance in-use stock was estimated by combining appliances demand per floor space ($ADP_p(t)$, in unit/floor area) and total residential floor space. Appliances demand per housing unit was estimated based on 2015 Residential Energy Consumption Survey (RECS)[5] and was kept constant for future projections (Table S1). This paper chose the following copper-intensive appliance types as major appliances for detailed analysis based on CI results (heat equipment is embedded in buildings): individual air conditioner, air cooler (an evaporative or swamp cooler), clothes washing machine, clothes dryer, dish washer, refrigerator, television (TV), computer screen, desktop without screen, laptop, microwave and cookstove.

$$IUS_{p-A}(t) = SL(t) * POP(t) * ADP_p(t) \quad (2)$$

As residential buildings and appliances have significantly different lifetimes (*Table S1*), their flows need to be assessed separately. Annual demand for residential buildings were estimated based on the building types, cohorts and lifetime distributions[1,23–25]. The appliance lifetime distributions were adopted from Wang et al.[4]. Appliance age file of 2015 was estimated from the Residential Energy Consumption Survey (RECS). No age information was available for TV, microwave, air cooker, computer related products and cookstove, thus we estimated cohorts by assuming that same number of appliances entered the market from previous years. By combining with appliances lifetime distribution, we calculated annual demand for appliances.

Annual inflow in year t of product p ($AI_p(t)$) includes two parts: annual in-use stock increasing and outflow replacement of all previous years. Annual in-use stock increasing (IUSI) from year t to year (t-1) of a product (p) was calculated as follows:

$$IUSI_p(t) = IUS_p(t) - IUS_p(t-1) \tag{3}$$

where $IUS_p(t)$ includes both $IUS_{p-FS}(t)$ and $IUS_{p-A}(t)$.

For residential buildings, archetypes were differentiated by multiplying $IUSI_p(t)$ with $AS(t)$ where $AS(t)$ represents archetype split among SFH, MFH and others in year t varying by scenarios as described in the Resource Efficiency and Climate Change (RECC) framework[7,9,23,25,26].

Outflow replacement of product p of all previous years in year t ($OR_p(t)$) was determined by summing up the outflow in year t from all the age-cohorts. The earliest years for appliances and buildings were set to be 1990 and 1900, respectively. $SF_p(t', t)$ represents the proportion of product p purchased in year t' that was still in-use in year t, and was calculated using its shape parameter k_p and scale parameter λ_p in *Table S1*.

$$SF_p(t', t) = e^{-((t-t')/\lambda_p)^{k_p}} \tag{4}$$

$Cohort_p(t')$ was the original inflow cohort of p in a previous year t' ($t' \leq t$). For convenience, we at first derived the cohorts of p before 2015 by using its 2015 age file and its Survival function (SF). Based on the survival amount of inflow p from previous year t' in 2015 ($SA_p(t', 2015)$) in the age file, $Cohort_p(t')$ was calculated below.

$$Cohort_p(t') = SA_p(t', 2015)/SF_p(t', 2015),\ t' \leq 2015 \tag{5}$$

The cohort of p after 2015 was the same as total annual inflow:

$$Cohort_p(t') = AI_p(t'),\ t' > 2015 \tag{6}$$

The outflow of p from a previous year t' in year t ($OR_p(t', t)$) was calculated as follows:

$$OR_p(t', t) = \begin{cases} Cohort_p(t',t) * (SF_p(t', (t-1)) - SF_p(t', t)), & t' < t \\ 0, & t' = t' \end{cases} \tag{7}$$

The total outflow of p from all previous years:

$$OR_p(t) = \sum_{t' = 1990}^{t} OR_p(t', t) \tag{8}$$

Annual inflow in year t of product p was then calculated as follows:

$$AI_p(t) = IUSI_p(t) + OR_p(t) \tag{9}$$

Copper in-use stock of product p for housing service, $Cu_{IUS_p(t)}$:

$$Cu_{IUS_p(t)} = IUS_p(t) * CC_p \tag{10}$$

where CC_p represents the copper content per product p. See S1-2 for copper intensity.

Annual copper demand due to production of product p for housing service, $Cu_{Pro_p(t)}$:

$$Cu_{Pro_p(t)} = AI_p(t) * TCR_p \tag{11}$$

where TCR_p represents the total copper requirement per product p. See S1-2 for copper intensity.

Annual copper demand due to building maintenance of existing stock in forms of home improvement, $Cu_{main_p(t)}$:

$$Cu_{main_p(t)} = IUS_p(t) * TCR_{main} \tag{12}$$

where TCR_{main} represents the total copper requirement to maintain existing product. Here we consider the home improvement for SFH, MFH and other residential building types. See S1-2 for copper intensity.

Total annual copper demand for capital formation and maintenance of in-use stock of product supporting housing service, $Cu_{TotDemand(t)}$:

$$Cu_{TotDemand(t)} = \sum_{p} (Cu_{Pro_p(t)} + Cu_{main_p(t)}) \tag{13}$$

EoL scrap generation from product p, $Cu_{EoL_p(t)}$:

$$Cu_{EoL_p(t)} = OR_p(t) * CC_p \tag{14}$$

Manufacturing scrap from product p, $Cu_{MS_p(t)}$:

$$Cu_{MS_p(t)} = \left(AI_p(t) * (TCR_p - CC_p) + IUS_p(t) * (TCR_{main} - CC_{main})\right) * PortionMS \tag{15}$$

where CC_p represents the copper content per product p, CC_{main} represents the copper content to maintain existing product, $PortionMS$ represents the portion in (TCR-CC) that is manufacturing scrap. According to Wang et al.[27], 66.7% and 86.5% of of the difference between TCR and CC are manufacturing scrap for residential building sectors and appliance sectors, respectively.

Scrap due to maintenance copper replacement for product p, $Cu_{MR_p(t)}$:

$$Cu_{MR_p(t)} = IUS_p(t) * CC_{main} \tag{16}$$

Total annual copper scrap generation:

$$Cu_{TotScrap(t)} = \sum_{p} (Cu_{EoL_p(t)} + Cu_{MS_p(t)} + Cu_{MR_p(t)}) \tag{17}$$

S-5. Potential RIR and demand gaps/scrap surplus

Minimal scrap surplus, $ScrapSurplus_{min}(t)$:

$$ScrapSurplus_{min}(t) = \begin{cases} 0, \\ if \quad (Cu_{TotScrap(t)} + ScrapSurplus_{min}(t-1) < Cu_{TotDemand(t)} \\ (Cu_{TotScrap(t)} + ScrapSurplus_{min}(t-1)) - Cu_{TotDemand(t)}, \\ if \quad (Cu_{TotScrap(t)} + ScrapSurplus_{min}(t-1) \geq Cu_{TotDemand(t)} \end{cases} \quad (18)$$

where $ScrapSurplus_{min}(t-1)$ is the leftover of scrap from year (t-1) if total scrap available was more than total copper demand in year (t-1).

Maximal scrap use, $ScrapUse_{max}(t)$:

$$ScrapUse_{max}(t) = \begin{cases} Cu_{TotScrap(t)} + ScrapSurplus_{min}(t-1), \\ if \quad (Cu_{TotScrap(t)} + ScrapSurplus_{min}(t-1) < Cu_{TotDemand(t)} \\ Cu_{TotDemand(t)}, \\ if \quad (Cu_{TotScrap(t)} + ScrapSurplus_{min}(t-1) \geq Cu_{TotDemand(t)} \end{cases} \quad (19)$$

Demand gap (minimal primary copper demand), $DemandGap_{min}(t)$:

$$DemandGap_{min}(t) = \begin{cases} Cu_{TotDemand(t)} - (Cu_{TotScrap(t)} + ScrapSurplus_{min}(t-1)), \\ if \quad (Cu_{TotScrap(t)} + ScrapSurplus_{min}(t-1) < Cu_{TotDemand(t)} \\ 0, \\ if \quad (Cu_{TotScrap(t)} + ScrapSurplus_{min}(t-1) \geq Cu_{TotDemand(t)} \end{cases} \quad (20)$$

The potential maximum RIR was estimated as the ratio of total copper scrap available and total copper demand, $RIR_{max}(t)$:

$$RIR_{max}(t) = \begin{cases} (Cu_{TotScrap(t)} + ScrapSurplus_{min}(t-1))/Cu_{TotDemand(t)}, \\ if \quad (Cu_{TotScrap(t)} + ScrapSurplus_{min}(t-1) < Cu_{TotDemand(t)} \\ 1, \\ if \quad (Cu_{TotScrap(t)} + ScrapSurplus_{min}(t-1) \geq Cu_{TotDemand(t)} \end{cases} \quad (21)$$

S-6. Tradeoffs due to in-use electricity use

The energy efficiency (EE) by ages were estimated using information from U.S. Energy Information Administration (EIA)[28] (Table S3 and Figure S1).

Table S3 Share of refrigerator shipments by maximum annual energy use (MAEU) and weighted average values

Year	Share of refrigerator shipments by MAEU in kWh/yr calculated from EIA[28]								Estimated weighted average MAEU (kWh/yr)
	713-571	<=570	528-477	<=476	528-450	<=449	528-423	<=422	
1998	80.62%	19.38%							628
1999	74.72%	25.28%							624
2000	72.88%	27.12%							622
2001			82.70%	17.30%					498
2002			79.93%	20.07%					497
2003			74.35%	25.65%					496
2004					66.78%	33.22%			476
2005					67.07%	32.93%			476
2006					68.84%	31.16%			477
2007					69.88%	30.12%			477
2008							69.15%	30.85%	459
2009							65.47%	34.53%	457
2010							50.00%	50.00%	449
2011							44.24%	55.76%	446
2012							23.85%	76.15%	435

Due to data availability, weighted averages of maximum annual energy use were calculated for year 1998~2012 (last column in Table S3) and estimated for past (1990~1997) and future (2013~2100) refrigerator sales in all years by fitting and extrapolating the EE improvement trend by year (Figure S1). According to this trend, the EE declines exponentially to 47 kWh/yr by 2100 which is hard but possible to achieve for small refrigerators (50L)[29]. This EE improvement situation was denoted as "EE ambitious improvement" in this paper. For comparison, two additional situations were analyzed: (a) EE no improvement - the weighted average of EE of

refrigerator sales does not change after 2012, and the values for 1990~1997 follow the minimum efficiency standards[28]; (b) EE conservative improvement – the weighted average of EE of refrigerator sales improves (annual electricity consumption declines) to 397 kWh/yr (the energy star standard in 2014[28]) by 2100, and the values for 1990~1997 follow the minimum efficiency standards[28].

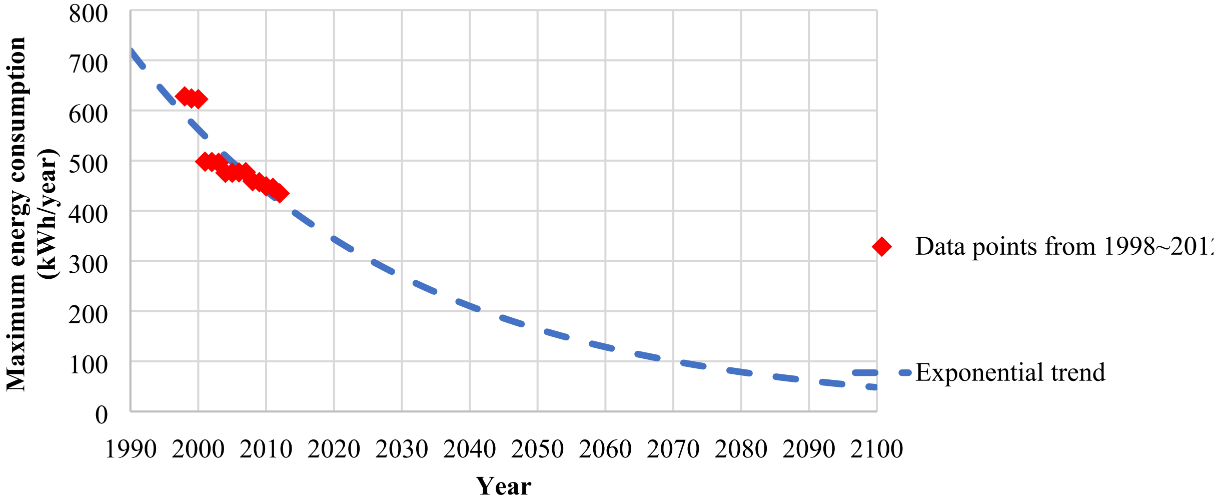

Figure S1 Fitting energy efficiency improvement by year

TCR for global refrigerators production and low voltage electricity generation in the US were assessed by life cycle assessment (LCA) using ecoinvent 3.6 cut-off database[19] to be 1404 g/unit and 0.234 g/kWh, respectively; similarly, global warming potential (GWP) by applying the IPCC 2013 100a characterization method were 272 kg CO2-eq/unit and 0.586 kg CO2-eq/kWh, respectively. TCR and GWP for refrigerators were kept constant for all assessed years. To assess the impact of CI increase in electricity generation in an anticipated renewable future[30–33], two situations were compared: (a) Electricity TCR no change – TCR per kWh of electricity generation keeps constant through the century; (b) Electricity TCR increase – TCR per kWh of

electricity generation increases linearly from 0.234 g/kWh in 2015 to 0.640 g/kWh in 2100 (0.640 g/kWh is the amount to generate one kWh of low voltage electricity using photovoltaic. Calculated using ecoinvent 3.6[19]). To assess the impact of greenhouse gas (GHG) emissions intensity decline for electricity generation, two situations were compared: (a) Electricity GHG no change – GHG per kWh of electricity generation keeps constant through the century; (b) Electricity GHG decline – GHG per kWh of electricity generation declines linearly to 0 by 2100 (this makes an annual decline rate of 6.9 g/kWh which is comparable to the estimated decline rate of 6.4 g/kWh for US electricity CO2 generation in the mid-case/business-as-usual scenario by National Renewable Energy Laboratory (NREL)[34]).

To show the changes of in-use stock of refrigerators by EE classes under different scenarios, EE was grouped into 7 groups based on the estimated MAEU: 0~100, 101~200, 201~300, 301~400, 401~500, 501~600 and >600 kWh/yr. For example, if refrigerator EE of year A was categorized into EE group B, then the market share of EE group B is 1 in year A. In this way, market share of energy efficiency matrix ($MSEE$: matrix dimension: 7*111) of refrigerators were identified. There are 7 rows representing 7 EE groups considered and 111 columns representing the age cohorts (1990~2100) of in-use machine in $MSEE$.

Share of energy efficiency classes of the in-use stock of refrigerators, $IUEE$:

$$IUEE = MSEE * AFWM \tag{22}$$

where $AFWM$ is the age file of refrigerator, matrix dimension: 111*85. There are 85 columns representing the years (2016~2100) when in-use electricity of refrigerators was assessed.

$$EleUse = EleIn * AFWM \tag{23}$$

where $EleIn$ represents the weighted annual electricity use for all years, matrix dimension: 1*111. $EleUse$ were estimated for base case scenario and lifetime extension scenarios separately.

At last, the copper saving and reduced environmental impact due to lifetime extension-induced less production demand was compared with the additional copper demand and environmental impact due to more electricity use; net effect was shown (Figure S2).

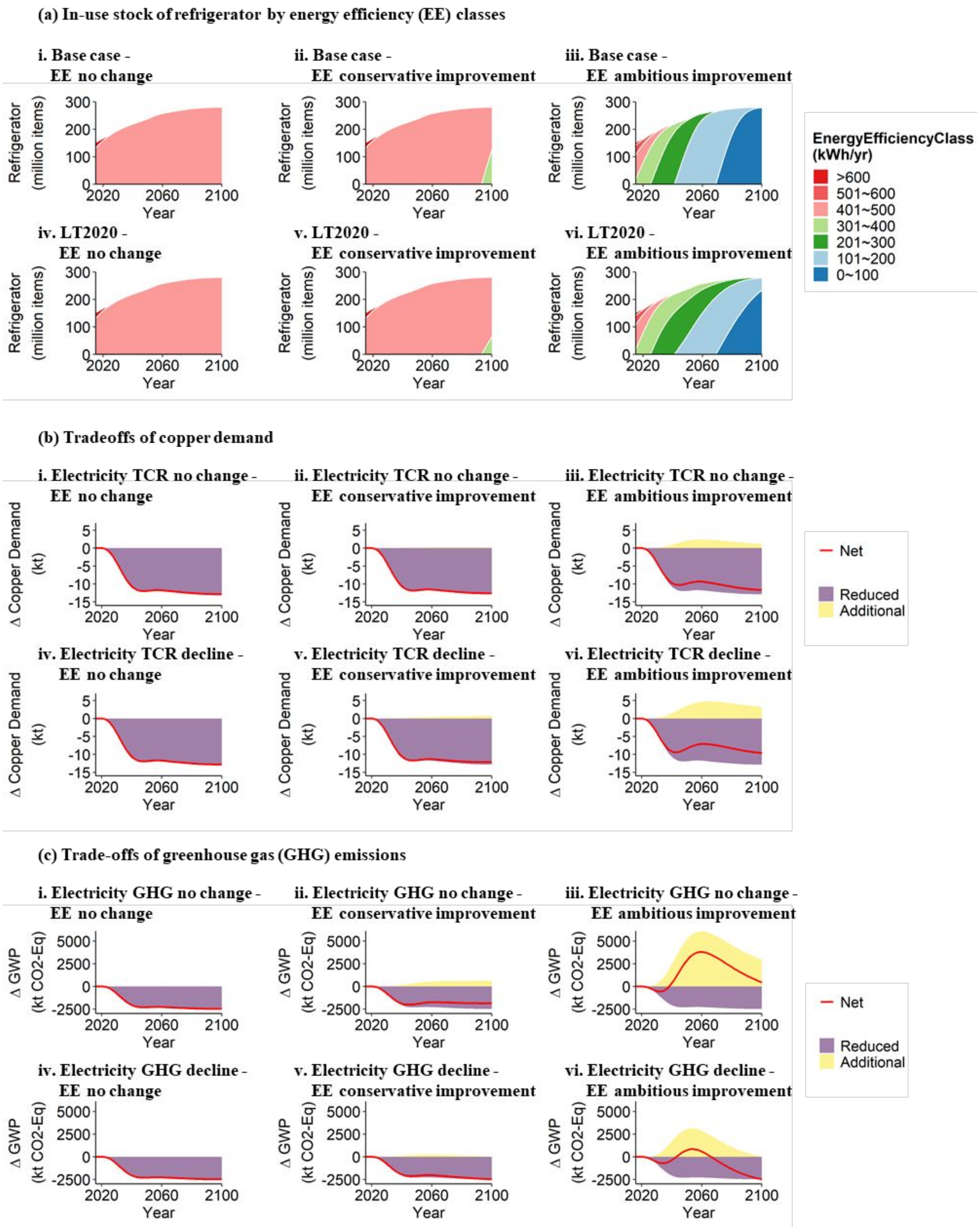
(a) In-use stock of refrigerator by energy efficiency (EE) classes
i. Base case - EE no change
ii. Base case - EE conservative improvement
iii. Base case - EE ambitious improvement
iv. LT2020 - EE no change
v. LT2020 - EE conservative improvement
vi. LT2020 - EE ambitious improvement
Refrigerator (million items)
Year
EnergyEfficiencyClass (kWh/yr)
>600
501~600
401~500
301~400
201~300
101~200
0~100
(b) Tradeoffs of copper demand
i. Electricity TCR no change - EE no change
ii. Electricity TCR no change - EE conservative improvement
iii. Electricity TCR no change - EE ambitious improvement
iv. Electricity TCR decline - EE no change
v. Electricity TCR decline - EE conservative improvement
vi. Electricity TCR decline - EE ambitious improvement
Δ Copper Demand (kt)
Year
Net
Reduced
Additional
(c) Trade-offs of greenhouse gas (GHG) emissions
i. Electricity GHG no change - EE no change
ii. Electricity GHG no change - EE conservative improvement
iii. Electricity GHG no change - EE ambitious improvement
iv. Electricity GHG decline - EE no change
v. Electricity GHG decline - EE conservative improvement
vi. Electricity GHG decline - EE ambitious improvement
Δ GWP (kt CO2-Eq)
Year
Net
Reduced
Additional

Figure S2 Lifetime extension-induced change of energy efficiency (EE) class composition, and copper demand and environmental impact related with refrigerator demand to fulfil US housing service. (a) Comparison of in-use stock of refrigerator by EE classes between base case((a)-i, ii and iii) and lifetime extension scenario((a)-iv, v, iv) under three different EE improvement situations. (b) Trade-offs in copper demand between reduced production and additional electricity consumption by less efficient refrigerators under different EE improvement and electricity total copper requirement (TCR) situations. (c) Trade-offs in greenhouse gas (GHG) emissions between reduced production and additional electricity consumption under different EE improvement and electricity GHG situations.

S-7. Products in-use stock, annual new demand and annual end-of-life demolish for housing services

In the stock-driven dynamic material flow analysis model, we multiply population and service level (floor area per capita) to generate required total in-use stock of residential floor area as shown in Figure S3. In SL2020, service level is set to be stable after 2020 (Figure S3 a.(ii)) where a relatively sharp turning point exists. For the SL_LED strategy (Figure S3 b.), the quickly decreasing service level offsets the increase of population and drives the steep decline of total in-use stock of floor area, until the service level stables after 2060 and a sharp turning point occurs.

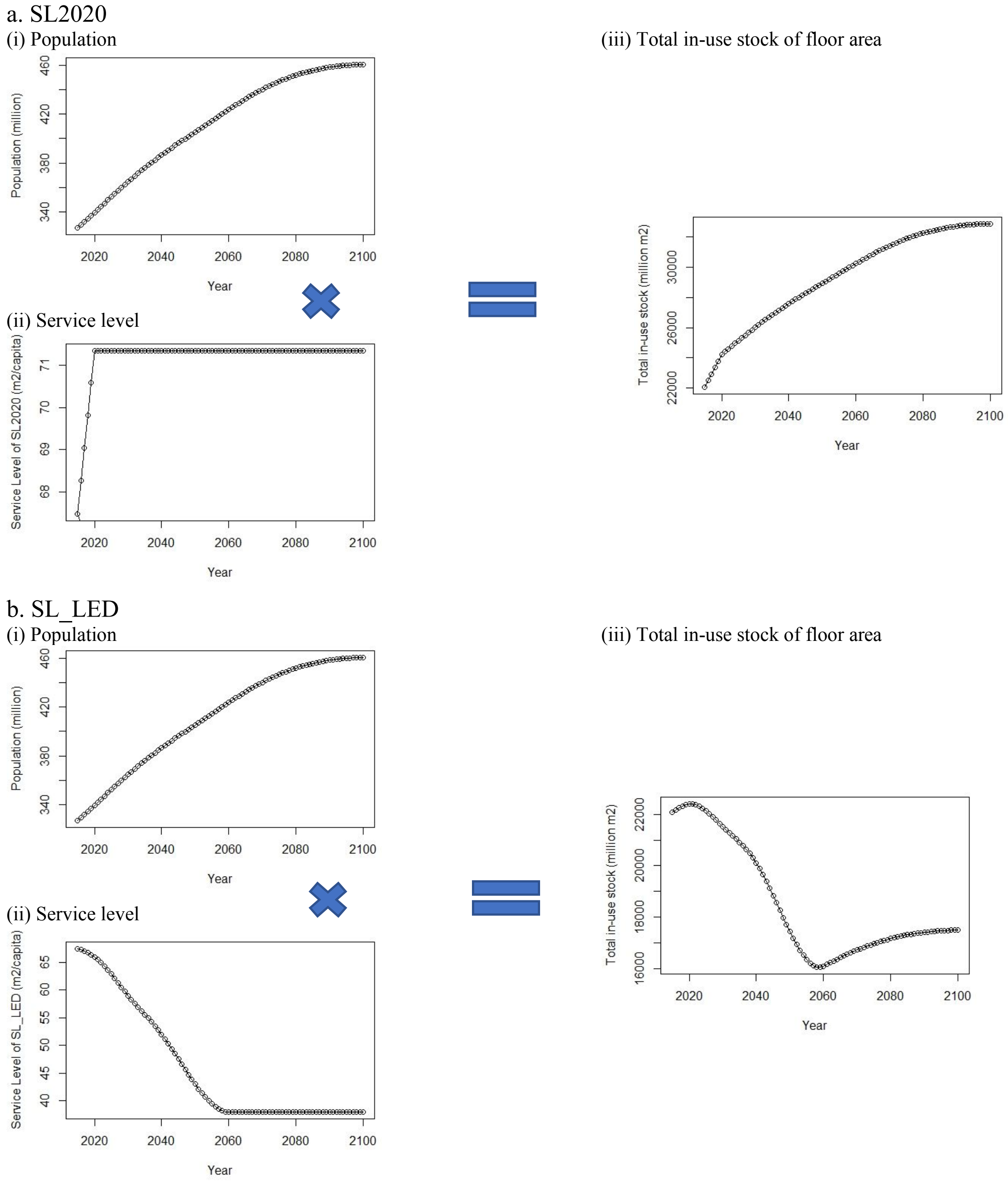

Figure S3 Generating total in-use stock of residential floor area

The results of in-use stock of products, annual demand of new products, and annual end of life (EoL) products providing housing services were shown in Figure S4, S5 and S6, respectively.

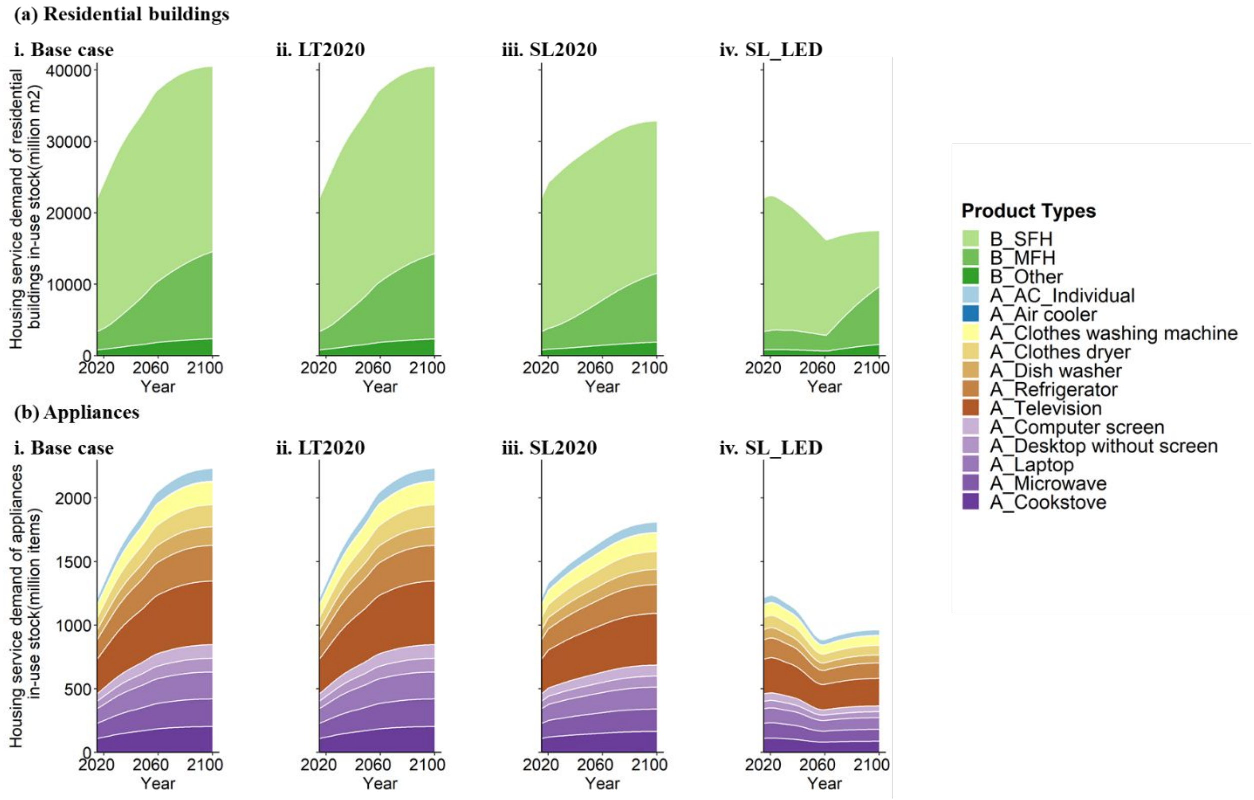

Figure S4 In-use stock of products for housing services under scenarios. (a) represents the in-use stock of residential buildings providing housing services. (b) represents the in-use stock of appliances providing housing services. LT2020 represents Strategy 1 - lifetime extension after 2020. SL2020 represents Strategy 2 - service level stable after 2020. SL_LED represents Strategy 3 - service level following the Low Energy Demand scenario[11,23]. SFH means single family residential building. MFH means multifamily residential building. Other means other residential structures. AC represents air conditioner. B- means building category. A_ means appliance category.

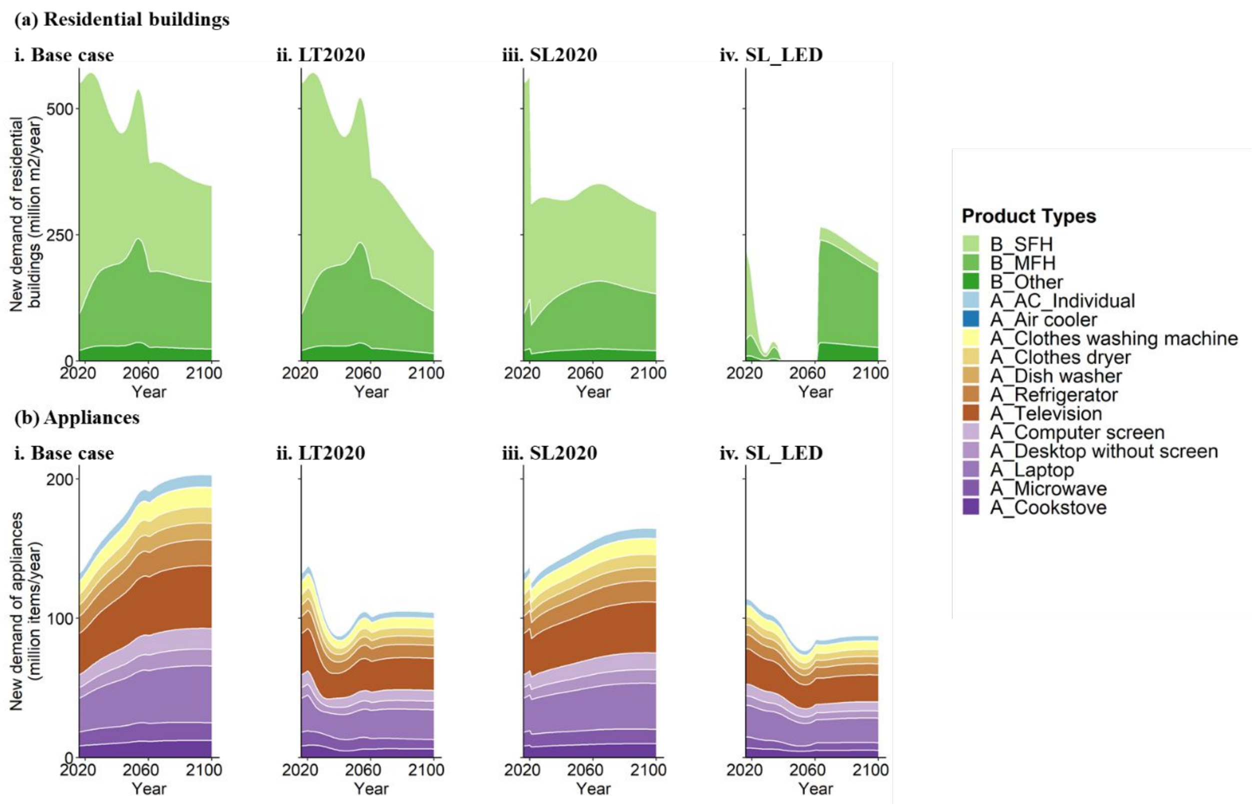

Figure S5 Annual demand of new products for housing services under scenarios. (a) represents new construction demand of residential buildings. (b) represents demand of new appliances. LT2020 represents Strategy 1 - lifetime extension after 2020. SL2020 represents Strategy 2 - service level stable after 2020. SL_LED represents Strategy 3 - service level following the Low Energy Demand scenario[11,23]. SFH means single family residential building. MFH means multifamily residential building. Other means other residential structures. AC represents air conditioner. B- means building category. A_ means appliance category.

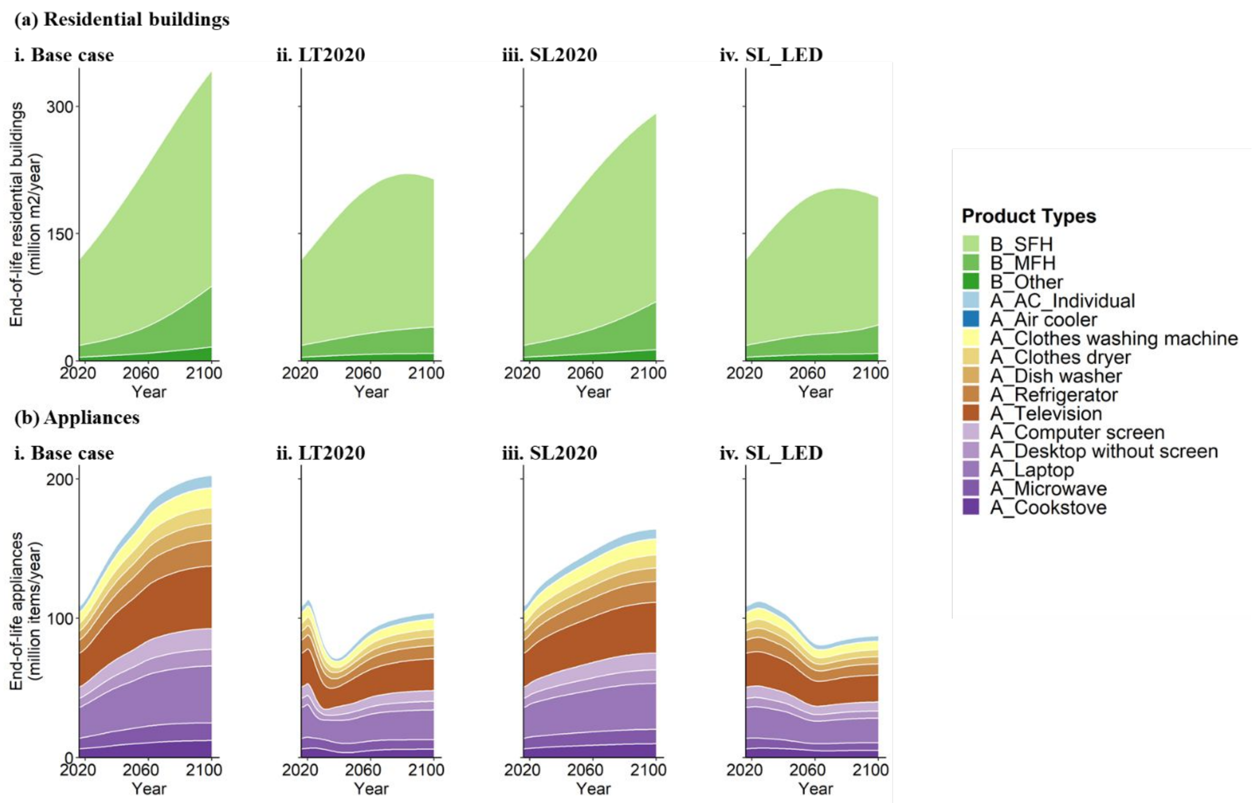

Figure S6 Annual end of life (EoL) products providing housing services under scenarios. (a) represents EoL residential buildings. (b) represents EoL appliances. LT2020 represents Strategy 1 - lifetime extension after 2020. SL2020 represents Strategy 2 - service level stable after 2020. SL_LED represents Strategy 3 - service level following the Low Energy Demand scenario[11,23]. SFH means single family residential building. MFH means multifamily residential building. Other means other residential structures. AC represents air conditioner. B- means building category. A_ means appliance category.

S-8. Glossary in alphabetic order

AC: Air conditioner.

BEA: Bureau of Economic Analysis.

CC: Copper content.

CE: Circular economy.

CFL: Compact fluorescent lamp.

CI: Copper intensity.

dMFA: Dynamic material flow analysis.

EE: Energy efficiency.

EIA: Energy Information Administration.

EoL: end-of-life.

EoL-RIR: End-of-life recycling rate. Portion of metal produced from end-of-life scrap.

GHG: Greenhouse gas.

GWP: Global warming potential.

HE: Heat equipment.

Housing service: Service provided by residential building stock and major household appliances.

LCA: Life cycle assessment.

LED: Low Energy Demand storyline.

LT2020: Lifetime extension after 2020.

ME: Material efficiency.

MFH: multi-family home.

MR: maintenance replacement.

MS: manufacturing scrap.

NREL: National Renewable Energy Laboratory.

RECC: Resource Efficiency and Climate Change framework.

RECS: Residential Energy Consumption Survey.

RIR: Recycling input rate. Portion of metal produced from scrap.

SFH: single-family home.

SL2020: Service level stable after 2020.

SL_LED: Service level following the Low Energy Demand storyline.

SSP: Socioeconomic pathways.

TCR: Total copper requirement.

WIO-MFA: waste input-output material flow analysis.

References

(1) Aktas, C. B.; Bilec, M. M. Impact of Lifetime on US Residential Building LCA Results. *Int J Life Cycle Assess* **2012**, *17* (3), 337–349. https://doi.org/10.1007/s11367-011-0363-x.

(2) Ianchenko, A.; Simonen, K.; Barnes, C. Residential Building Lifespan and Community Turnover. *Journal of Architectural Engineering* **2020**, *26* (3), 04020026. https://doi.org/10.1061/(ASCE)AE.1943-5568.0000401.

(3) Miatto, A.; Schandl, H.; Tanikawa, H. How Important Are Realistic Building Lifespan Assumptions for Material Stock and Demolition Waste Accounts? *Resources, Conservation and Recycling* **2017**, *122*, 143–154. https://doi.org/10.1016/j.resconrec.2017.01.015.

(4) Wang, F.; Huisman, J.; Stevels, A.; Baldé, C. P. Enhancing E-Waste Estimates: Improving Data Quality by Multivariate Input–Output Analysis. *Waste Management* **2013**, *33* (11), 2397–2407. https://doi.org/10.1016/j.wasman.2013.07.005.

(5) Residential Energy Consumption Survey (RECS) - Data - U.S. Energy Information Administration (EIA) https://www.eia.gov/consumption/residential/data/2015/#house (accessed 2020 -10 -18).

(6) B. Müller, D. Stock Dynamics for Forecasting Material Flows—Case Study for Housing in The Netherlands. *Ecological Economics* **2006**, *59* (1), 142–156. https://doi.org/10.1016/j.ecolecon.2005.09.025.

(7) IRP. *Resource Efficiency and Climate Change: Material Efficiency Strategies for a Low-Carbon Future*; United Nations Environment Programme: Nairobi, Kenya., 2020.

(8) Pauliuk, S.; Fishman, T.; Heeren, N.; Berrill, P.; Tu, Q.; Wolfram, P.; Hertwich, E. G. Linking Service Provision to Material Cycles: A New Framework for Studying the Resource Efficiency–Climate Change (RECC) Nexus. *Journal of Industrial Ecology* **2021**, *25* (2), 260–273. https://doi.org/10.1111/jiec.13023.

(9) Riahi, K.; Vuuren, D. P. van; Kriegler, E.; Edmonds, J.; O'Neill, B. C.; Fujimori, S.; Bauer, N.; Calvin, K.; Dellink, R.; Fricko, O.; Lutz, W.; Popp, A.; Cuaresma, J. C.; KC, S.; Leimbach, M.; Jiang, L.; Kram, T.; Rao, S.; Emmerling, J.; Ebi, K.; Hasegawa, T.; Havlik, P.; Humpenöder, F.; Silva, L. A. D.; Smith, S.; Stehfest, E.; Bosetti, V.; Eom, J.; Gernaat, D.; Masui, T.; Rogelj, J.; Strefler, J.; Drouet, L.; Krey, V.; Luderer, G.; Harmsen, M.; Takahashi, K.; Baumstark, L.; Doelman, J. C.; Kainuma, M.; Klimont, Z.; Marangoni, G.; Lotze-Campen, H.; Obersteiner, M.; Tabeau, A.; Tavoni, M. The Shared Socioeconomic Pathways and Their Energy, Land Use, and Greenhouse Gas Emissions Implications: An Overview. *Global Environmental Change* **2017**, *42*, 153–168. https://doi.org/10.1016/j.gloenvcha.2016.05.009.

(10) Fishman, T.; Heeren, N.; Pauliuk, S.; Berrill, P.; Tu, Q.; Wolfram, P.; Hertwich, E. G. A Comprehensive Set of Global Scenarios of Housing, Mobility, and Material Efficiency for Material Cycles and Energy Systems Modeling. *Journal of Industrial Ecology* **2021**, *25* (2), 305–320. https://doi.org/10.1111/jiec.13122.

(11) Grubler, A.; Wilson, C.; Bento, N.; Boza-Kiss, B.; Krey, V.; McCollum, D. L.; Rao, N. D.; Riahi, K.; Rogelj, J.; De Stercke, S.; Cullen, J.; Frank, S.; Fricko, O.; Guo, F.; Gidden, M.; Havlík, P.; Huppmann, D.; Kiesewetter, G.; Rafaj, P.; Schoepp, W.; Valin, H. A Low Energy Demand Scenario for Meeting the 1.5 °C Target and Sustainable Development Goals without Negative Emission Technologies. *Nature Energy* **2018**, *3* (6), 515–527. https://doi.org/10.1038/s41560-018-0172-6.

(12) Wang, T.; Berrill, P.; Zimmerman, J. B.; Hertwich, E. G. Copper Recycling Flow Model for the United States Economy: Impact of Scrap Quality on Potential Energy Benefit. *Environ. Sci. Technol.* **2021**. https://doi.org/10.1021/acs.est.0c08227.

(13) U.S. Census Bureau. American Housing Survey (AHS) https://www.census.gov/programs-surveys/ahs.html (accessed 2020 -10 -18).

(14) Bureau of Economic Analysis (BEA). Industry Economic Account Data: GDP by Industry https://apps.bea.gov/iTable/iTable.cfm?reqid=150&step=3&isuri=1&table_list=240&categories=ugdpxind (accessed 2021 -07 -31).

(15) Heeren, N.; Fishman, T. A Database Seed for a Community-Driven Material Intensity Research Platform. *Scientific Data* **2019**, *6* (1), 23. https://doi.org/10.1038/s41597-019-0021-x.

(16) Dong, D.; Tukker, A.; Van der Voet, E. Modeling Copper Demand in China up to 2050: A Business-as-Usual Scenario Based on Dynamic Stock and Flow Analysis. *Journal of Industrial Ecology* **2019**, *23* (6), 1363–1380. https://doi.org/10.1111/jiec.12926.

(17) Marinova, S.; Deetman, S.; van der Voet, E.; Daioglou, V. Global Construction Materials Database and Stock Analysis of Residential Buildings between 1970-2050. *Journal of Cleaner Production* **2020**, *247*, 119146. https://doi.org/10.1016/j.jclepro.2019.119146.

(18) Deetman, S.; Pauliuk, S.; van Vuuren, D. P.; van der Voet, E.; Tukker, A. Scenarios for Demand Growth of Metals in Electricity Generation Technologies, Cars, and Electronic Appliances. *Environmental Science & Technology* **2018**, *52* (8), 4950–4959. https://doi.org/10.1021/acs.est.7b05549.

(19) ecoinvent https://www.ecoinvent.org/ (accessed 2020 -01 -05).

(20) Berrill, P.; Miller, T. R.; Kondo, Y.; Hertwich, E. G. Capital in the American Carbon, Energy, and Material Footprint. *Journal of Industrial Ecology* **2020**, *24* (3), 589–600. https://doi.org/10.1111/jiec.12953.

(21) Miller, T. R.; Berrill, P.; Wolfram, P.; Wang, R.; Kim, Y.; Zheng, X.; Hertwich, E. G. Method for Endogenizing Capital in the United States Environmentally-Extended Input-Output Model. *Journal of Industrial Ecology* **2019**, *23* (6), 1410–1424. https://doi.org/10.1111/jiec.12931.

(22) NAICS Code: 333415 Air-Conditioning and Warm Air Heating Equipment and Commercial and Industrial Refrigeration Equipment Manufacturing https://www.naics.com/naics-code-description/?code=333415 (accessed 2020 -11 -20).

(23) Fishman, T.; Heeren, N.; Pauliuk, S.; Berrill, P.; Tu, Q.; Wolfram, P.; Hertwich, E. *A Comprehensive Set of Global Scenarios of Housing, Mobility, and Material Efficiency for Material Cycles and Energy Systems Modelling*; preprint; SocArXiv, 2020. https://doi.org/10.31235/osf.io/tqsc3.

(24) Resource Efficiency and Climate Change | Resource Panel https://www.resourcepanel.org/reports/resource-efficiency-and-climate-change (accessed 2020 -10 -08).

(25) Pauliuk, S. *Documentation of Part IV of the RECC Model Framework: Open Dynamic Material Systems Model for the Resource Efficiency-Climate Change Nexus (ODYM-RECC), v2.2*; preprint; SocArXiv, 2020. https://doi.org/10.31235/osf.io/y4xcv.

(26) Hertwich, E.; Lifset, R.; Pauliuk, S.; Heeren, N.; Ali, S.; Tu, Q.; Ardente, F.; Berrill, P.; Fishman, T.; Kanaoka, K.; Kulczycka, J.; Makov, T.; Masanet, E.; Wolfram, P. *Resource Efficiency and Climate Change: Material Efficiency Strategies for a Low-Carbon Future*; Zenodo, 2019. https://doi.org/10.5281/zenodo.3542681.

(27) Wang, T.; Berrill, P.; Zimmerman, J. B.; Hertwich, E. G. Copper Recycling Flow Model for the United States Economy: Impact of Scrap Quality on Potential Energy Benefit. *Environ. Sci. Technol.* **2021**, *55* (8), 5485–5495. https://doi.org/10.1021/acs.est.0c08227.

(28) U.S. Energy Information Administration (EIA). Residential End Uses: Historical Efficiency Data and Incremental Installed Costs for Efficiency Upgrades. **2017**, 116.

(29) Park, W. Y.; Shah, N.; Phadke, A. Enabling Access to Household Refrigeration Services through Cost Reductions from Energy Efficiency Improvements. *Energy Efficiency* **2019**, *12* (7), 1795–1819. https://doi.org/10.1007/s12053-019-09807-w.

(30) Hertwich, E. G.; Gibon, T.; Bouman, E. A.; Arvesen, A.; Suh, S.; Heath, G. A.; Bergesen, J. D.; Ramirez, A.; Vega, M. I.; Shi, L. Integrated Life-Cycle Assessment of Electricity-Supply Scenarios Confirms Global Environmental Benefit of Low-Carbon Technologies. *PNAS* **2015**, *112* (20), 6277–6282. https://doi.org/10.1073/pnas.1312753111.

(31) Kleijn, R.; van der Voet, E. Resource Constraints in a Hydrogen Economy Based on Renewable Energy Sources: An Exploration. *Renewable and Sustainable Energy Reviews* **2010**, *14* (9), 2784–2795. https://doi.org/10.1016/j.rser.2010.07.066.

(32) Pehnt, M.; Oeser, M.; Swider, D. J. Consequential Environmental System Analysis of Expected Offshore Wind Electricity Production in Germany. *Energy* **2008**, *33* (5), 747–759. https://doi.org/10.1016/j.energy.2008.01.007.

(33) Harmsen, J. H. M.; Roes, A. L.; Patel, M. K. The Impact of Copper Scarcity on the Efficiency of 2050 Global Renewable Energy Scenarios. *Energy* **2013**, *50*, 62–73. https://doi.org/10.1016/j.energy.2012.12.006.

(34) National Renewable Energy Laboratory (NREL). Cambium | Standard Scenarios 2020 | All Scenarios (Annual data only) https://cambium.nrel.gov/?project=c3fec8d8-6243-4a8a-9bff-66af71889958 (accessed 2021 -07 -27).
